# Autophagy inhibition in intestinal stem cells favors enteroendocrine cell differentiation through Stat92E activity

**DOI:** 10.1242/dmm.052214

**Published:** 2025-12-29

**Authors:** Camille Lacarrière-Keïta, Sonya Nassari, Jessica Boutet, Véronique Gaudreault, Steve Jean

**Affiliations:** Faculté de Médecine et des Sciences de la Santé, Départment d'immunologie et de biologie cellulaire, Université de Sherbrooke, 3201, Rue Jean Mignault, Sherbrooke, QC J1E 4K8, Canada

**Keywords:** Autophagy, Intestinal stem cell, Differentiation, *Drosophila*, JAK-STAT, Cell fate, Stat92E, Scute

## Abstract

Because the intestinal epithelium is exposed to various stressors, dysregulation of essential mechanisms that maintain gut homeostasis, such as autophagy, has been linked to inflammatory bowel pathologies. In *Drosophila melanogaster*, inhibition of autophagy specifically in adult intestinal stem cells (ISCs) affects their proportions differently during aging. Proper intestinal renewal requires a balance between ISC proliferation and differentiation. Here, we showed that, in adult ISCs, loss of core autophagy genes and regulators of autophagosome–lysosome fusion increases the enteroendocrine cell population and enhances the transcriptional activity of Stat92E. Functional experiments involving cell fate regulators of enteroendocrine or enterocyte differentiation and proliferation suggested that dysfunctional autophagy in adult ISCs enhances Stat92E activity downstream of Hop/JAK kinase. Finally, lineage-tracing analyses confirmed that autophagy inhibition promotes enteroendocrine cell differentiation. Thus, our data demonstrate that, under homeostatic conditions, basal autophagy limits enteroendocrine cell differentiation by regulating Stat92E activity, which can be counteracted by the transcription factor Scute.

## Teaser

**Summary:** Autophagy inhibition in *Drosophila* intestinal stem cells increases enteroendocrine cell differentiation in a Stat92E- and Scute-dependent manner.

## INTRODUCTION

The intestinal tract faces multiple risks of tissue injury caused by food or pathogen ingestion and proximity to the microbiota (Funk et al., 2020). Therefore, even under homeostatic conditions, rapid intestinal epithelium renewal is paramount to ensure adequate food digestion, nutrient absorption and barrier function (Funk et al., 2020). Intestinal cell turnover relies on intestinal stem cell (ISC) capacity to replenish damaged tissues with a definite proportion of nutrient-absorbing and secretory cells (Beumer and Clevers, 2021). External cues and inherent properties tightly regulate the fate of ISCs by balancing their proliferation and differentiation state to preserve tissue functions (Beumer and Clevers, 2021). Dysregulation of ISCs' regenerative capacity can favor the development of inflammatory bowel disease (IBD) (Graham and Xavier, 2020).

Genome-wide association studies of IBD susceptibility genes have identified numerous autophagy regulators, suggesting that autophagy plays an essential role in intestinal homeostasis (Henckaerts et al., 2011; Franke et al., 2010; Barrett et al., 2008). Accordingly, autophagy impairment in the intestinal epithelial cells leads to loss of barrier integrity, failure to eliminate intracellular pathogens, decrease in antimicrobial defense and microbiota dysbiosis (Nighot et al., 2015; Lassen et al., 2014; Cadwell et al., 2008; Bel et al., 2017; Patel et al., 2013; Larabi et al., 2019). Autophagy plays a vital role in the regenerative capacity of ISCs. Knockout (KO) of autophagy-related gene 5 (*Atg5*) in the intestinal epithelium of mice diminished the number of ISCs and reduced the intestinal organoid formation capacity *ex vivo* (Asano et al., 2017). Moreover, ISCs isolated from the intestinal epithelium of *Atg7* or *Atg16l1* KO mice displayed decreased enteroid survival (Trentesaux et al., 2020; Matsuzawa-Ishimoto et al., 2017). These observations support the requirement of autophagy for ISC function in mice. However, these results were obtained from a KO performed on all intestinal epithelial cell types, making it difficult to conclude an ISC-specific intrinsic role for autophagy.

*Drosophila melanogaster* has emerged as an invaluable tool to interrogate cell-specific roles of genes in intestinal functions (Capo et al., 2019), given the multitude of genetic tools and reporters available (del Valle Rodríguez et al., 2011). Notably, the signaling pathways regulating ISC proliferation and differentiation are conserved from *Drosophila* to mammals, with less redundancy in flies (Jasper, 2020). Recent studies have demonstrated that the long-term RNA interference (RNAi)-mediated depletion of diverse autophagy genes in ISCs and their progenitor cells induces ISC exhaustion via an apoptotic response to DNA damage accumulation (Nagy et al., 2018). This loss of ISCs is reminiscent of the phenotype observed in a pan-intestinal epithelium *Atg7* KO mouse model (Trentesaux et al., 2020). In contrast, short-term RNAi-mediated depletion of Atg genes in fly ISCs and their progenitor cells increases their proliferation through hyperactivation of the epidermal growth factor receptor (EGFR) pathway (Zhang et al., 2019). Hence, autophagy limits adult ISC proliferation in younger individuals and prevents premature apoptotic death during aging.

Autophagy may also influence intestinal cell differentiation. In mice lacking *Atg16l1*, the proportion of secretory Paneth cells among intestinal epithelial cells is reduced (Lassen et al., 2014; Matsuzawa-Ishimoto et al., 2017). Similarly, KO of *Atg14* or *FIP200* (also known as *Rb1cc1*) in the intestinal epithelium results in shortened villi (Jung et al., 2019). In *Drosophila*, deletion of *Atg101* leads to a decrease in absorptive enterocytes (Guo et al., 2019a). Despite these findings, no direct link has yet been established between autophagy and the regulation of intestinal cell differentiation. This is unexpected, given that autophagy is known to modulate both stemness and differentiation in other adult stem cell systems (Lacarrière-Keïta et al., 2022; Clarke and Simon, 2019). For instance, high basal autophagy levels help maintain the stemness of mouse hematopoietic stem cells (Warr et al., 2013). Conversely, conditional KO of *Atg7* in these cells blocks their differentiation into mature neutrophils (Riffelmacher et al., 2017), while deletion of *Atg5* in naïve CD4^+^ T cells promotes their commitment to the T_H_9 helper T-cell lineage (Rivera Vargas et al., 2017). In *Drosophila*, autophagy induction supports cyst cell self-renewal, whereas it suppresses differentiation of testis cyst cell progenitors (Sênos Demarco et al., 2020). As autophagy affects the regenerative capacity of ISCs, it is plausible that it also influences their differentiation. However, the mechanisms by which autophagy regulates ISC behavior remain poorly understood.

At the cellular level, autophagy is a highly conserved catabolic process that regulates the lysosomal degradation of intracellular components (Yamamoto et al., 2023). An array of Atg proteins orchestrates the formation of a double-membrane phagophore, resulting in its elongation and cargo incorporation, or by selectively surrounding the components interacting with an autophagy cargo adaptor, such as Ref(2)P (SQSTM1/p62 in mammals) (Devorkin and Gorski, 2014; Shatz and Elazar, 2024; Sakai et al., 2024). Ultimately, the phagophore seals and matures to form an autophagosome, fusing with lysosomes to degrade and recycle the sequestered constituents (Lőrincz and Juhász, 2019). Basal autophagic flux, the ratio of autophagosome formation to autolysosome degradation, can be either increased or repressed in response to environmental cues, by targeting, for example, Atg1/ULK1 activation (Galluzzi et al., 2018). Additionally, the fusion step requires coordinated actions between core Atg regulators and auxiliary proteins such as soluble *N*-ethylmaleimide-sensitive factor attachment protein receptors (SNAREs), tethering complexes and RAB GTPase proteins (Birgisdottir and Johansen, 2020; Zhao et al., 2021). Previous work from our laboratory has demonstrated the requirement for the endosomal small GTPase Rab21 in autophagosome–lysosome fusion (Jean et al., 2015) in flies and mammals (Lauzier et al., 2019; Nassari et al., 2022). Once activated by the guanine exchange factor set-binding factor (Sbf), Rab21 promotes the trafficking of vacuolar-associated membrane protein 7 (Vamp7) from early endosomes to lysosomes (Jean et al., 2015). Vamp7 in flies (and VAMP8 in mammals) is associated with synaptosomal-associated protein 29 (SNAP29) and autophagosomal Syntaxin 17 (Stx17) to induce autophagosome–lysosome membrane fusion (Itakura et al., 2012). VAMP8/Stx17/SNAP29 is one of the two SNARE complex combinations involved in autophagosome–lysosome fusion (Gao et al., 2018; Bas et al., 2018; Takáts et al., 2018; Matsui et al., 2018). Rab21 and VAMP8 have known functions in the gut, with Rab21 affecting nutrient-absorptive enterocyte survival in *Drosophila* (Nassari et al., 2022), and VAMP8 regulating mucus secretion by goblet cells in mice (Cornick et al., 2019). However, their roles in ISCs and their link to autophagy have not been explored in this context.

Here, we show that Rab21 loss and autophagy inhibition, specifically in adult ISCs, favor enteroendocrine cell differentiation. Mechanistically, a reduction in autophagic flux increases the activation of Signal transducer and activator of transcription 92E (Stat92E) by acting downstream of Hopscotch (Hop)/Janus kinase (JAK), causing an increase in the enteroendocrine cell population. Our data suggest that basal autophagy limits enteroendocrine cell differentiation by regulating Stat92E in adult ISCs.

## RESULTS

### Rab21 depletion in adult ISCs increases progenitor and mature enteroendocrine cell populations

**Fig. 1.**
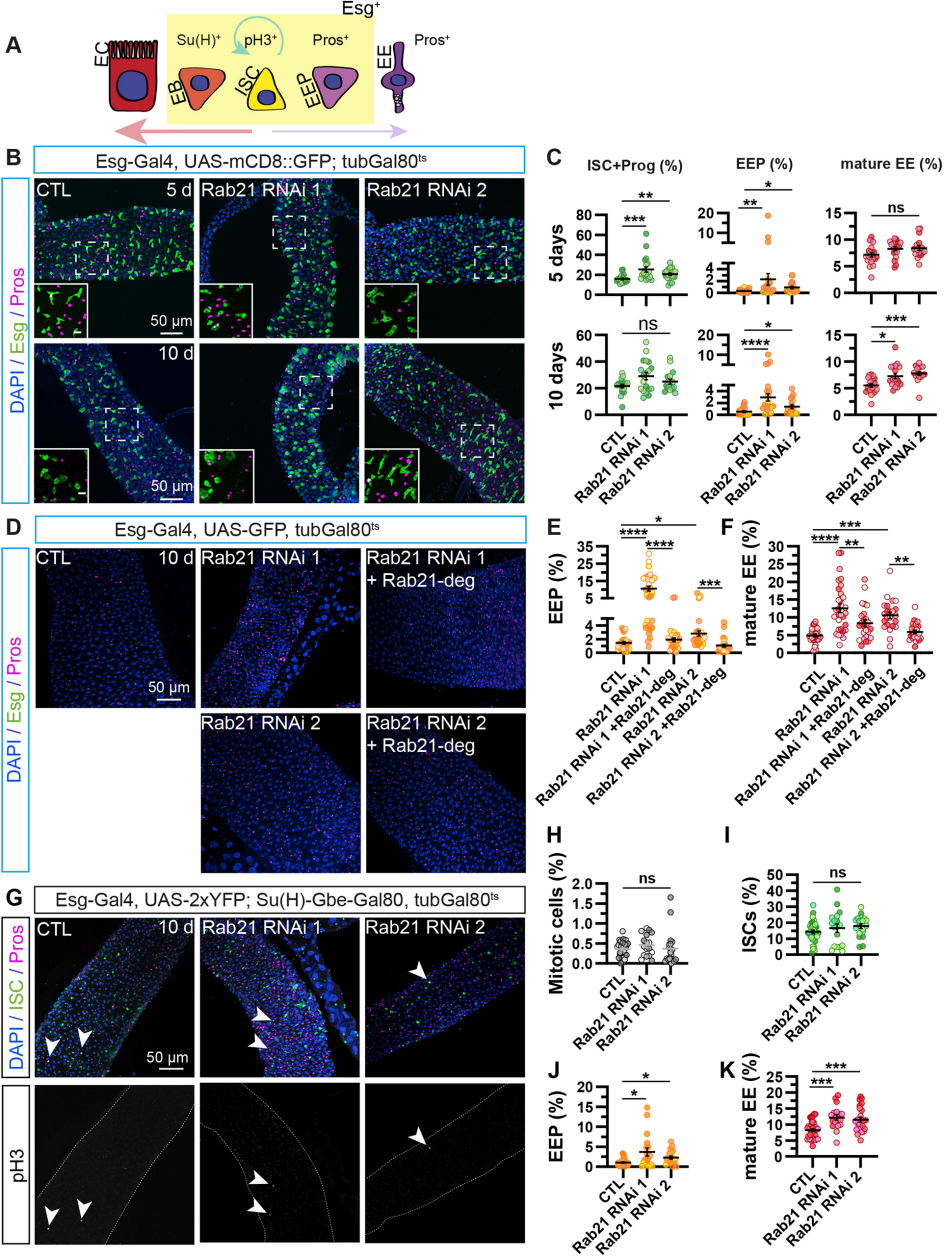
**Rab21 depletion in adult intestinal stem cells increases enteroendocrine cell populations.** (A) Schematic representation of intestinal cell lineages and cell-specific markers (Boumard and Bardin, 2021). The schematic was generated by C.L.-K. and is not a direct reproduction of previously published schematics. EB, enteroblast; EC, enterocyte; EE, enteroendocrine cell; EEP, enteroendocrine progenitor cell; Esg, Escargot; ISC, intestinal stem cell; ; pH3, phospho-histone 3; Pros, Prospero; Su(H), Suppressor of Hairless. (B,C) Adult *Drosophila* posterior midgut from *esg-Gal4*, *UAS-mCD8::GFP*; *tubGal80^ts^* driver expressing *UAS-LacZ* [control (CTL)], *Rab21* RNA interference (RNAi) 1 or *Rab21* RNAi 2 for 5 or 10 days, in ISCs and progenitors. (B) Representative maximal projections. GFP labels Esg^+^ ISCs and progenitor cells (green), anti-Pros antibody marks EEs (magenta), and DAPI stains nuclei. Scale bars: 50 µm; 10 μm (insets). (C) Quantification of the percentage of GFP^+^ ISC and progenitor cells (left column) over total cells (DAPI^+^); GFP^+^, Pros^+^ EEPs (middle column) over total cells (DAPI^+^); and GFP^−^, Pros^+^ mature EEs (right column) over total cells (DAPI^+^). After 5 days (upper row) or 10 days (lower row) of RNAi, *n*≥17guts. (D-F) Adult *Drosophila* posterior midgut from *esg-Gal4*, *UAS-GFP*, *tubGal80^ts^* driver expressing *UAS-LacZ* (CTL) and co-expressing *Rab21* RNAi 1 and 2 with *UAS-LacZ* or *UAS-Rab21*-deg for 10 days, in ISCs and progenitors. (D) Representative maximal projections. GFP labels Esg^+^ ISCs and progenitor cells (green) over total cells (DAPI^+^), anti-Pros antibody marks EEs (magenta), and DAPI stains nuclei. Scale bar: 50 µm. (E,F) Quantification of the percentage of GFP^+^, Pros^+^ EEPs (E) and GFP^−^, Pros^+^ mature EE cells (F); *n*≥21 guts. (G) Adult *Drosophila* posterior midgut from *esg-Gal4*, *UAS-2xYFP*; *Su(H)-GBE-Gal80, tubGal80^ts^* driver expressing *UAS-LacZ* (CTL), *Rab21* RNAi 1 or *Rab21* RNAi 2 for 10 days in ISCs. YFP labels ISCs (green), anti-Pros antibody marks EE cells (magenta), pH3 labels the mitotic ISCs, and DAPI stains nuclei. Scale bar: 50 µm. Representative maximal projections. Arrowheads indicate pH3^+^ cells. (H-K) Quantification of the percentage of pH3^+^ mitotic cells over total cells (DAPI^+^) (H); YFP^+^, Pros^−^ ISCs over total cells (DAPI^+^) (I); YFP^+^, Pros^+^ EEPs over total cells (DAPI^+^) (J); and YFP^−^, Pros^+^ mature EE cells over total cells (DAPI^+^) (K); *n*≥18 guts. *N*=3 independent experiments from three independent crosses. Quantifications represent the mean±s.e.m. Each dot represents an intestine. Kruskal–Wallis test was used, followed by Dunn's comparison tests. *P<0.05, **P<0.01, ***P<0.001, ****P<0.0001; ns, non-significant (*P*>0.05). Also see Fig. S1.

In *Drosophila*, ISCs divide into stem cells and progenitor cells, named enteroendocrine progenitor cells (EEPs) or enteroblasts, which differentiate into enteroendocrine cells or enterocytes, respectively (Guo et al., 2019b; Chen et al., 2018) (Fig. 1A). Data from a genome-wide RNAi screen targeting ISCs and progenitor cells using the Esg-Gal4^ts^ driver highlighted autophagic genes and *Rab21* as regulators of ISC and progenitor density (Zeng et al., 2015). We recently demonstrated roles for Rab21 in enterocytes, and showed preferential expression of this early endosome-enriched RAB GTPase in the copper cell and R5 regions of the posterior midgut (Nassari et al., 2022). Additionally, we detected its expression in all cell types of the R5 region, including delta-positive stem cells (Nassari et al., 2022).

Because autophagy inhibition affects ISCs differently depending on the knockdown duration (Nagy et al., 2018; Zhang et al., 2019), we investigated the requirement for Rab21, as a regulator of autophagy, in ISCs and progenitor cells in adult flies under homeostatic conditions. We used the targeting temporal and regional gene expression targeting (TARGET) system (McGuire et al., 2004), combined with the Esg-Gal4 driver, to deplete Rab21 using two previously validated independent RNAi constructs (Jean et al., 2015). Intestinal cell populations in the R5 midgut region were analyzed 5 or 10 days after RNAi induction (Fig. 1B,C). We calculated percentages of each cell population by automated image analysis of labeled-specific cells normalized to the total number of cells (DAPI^+^) for all images, as previously done by others (Okumura et al., 2016; Bardin et al., 2010; Loza-Coll et al., 2014; Biteau and Jasper, 2014; Ho et al., 2022). Green fluorescent protein (GFP) was expressed in Esg^+^ ISCs and progenitors (Fig. 1A) (Korzelius et al., 2014). Consistent with former observations (Zeng et al., 2015), the depletion of Rab21 significantly augmented the percentage of GFP^+^ ISCs and progenitor cells after 5 days of RNAi induction (Fig. 1B,C). However, the percentage of GFP^+^ undifferentiated cells showed a tendency to increase 10 days after Rab21 depletion, but this change did not reach statistical significance (Fig. 1B,C). Unexpectedly, the percentage of total enteroendocrine cells labeled with Prospero (Pros) (Ohlstein and Spradling, 2006; Micchelli and Perrimon, 2006) increased after 5 and 10 days of knockdown (Fig. S1A). We quantified mature enteroendocrine cells (Pros^+^, GFP^−^) and their specific progenitors, EEPs (Pros^+^ and GFP^+^) (Korzelius et al., 2014). Five days after RNAi induction, Rab21 loss in ISCs and progenitor cells only induced higher levels of EEPs (Fig. 1B,C). Interestingly, the depletion of Rab21 for 10 days increased both EEP and mature enteroendocrine cell populations (Fig. 1B,C).

As ISCs undergo asymmetric division to give rise to new ISCs and progenitor cells, we wondered whether the expansion in EEPs decreased the formation of enteroblasts. Using the Esg-Gal4^ts^ driver, we co-expressed the Notch reporter Su(H)-GFP (Bardin et al., 2010) with *Rab21* RNAis to quantify Su(H)^+^ enteroblasts, and CFP^+^ ISCs and progenitor cells (O'Brien et al., 2011). Depletion of Rab21 for 10 days did not affect the ratio of Su(H)^+^ enteroblasts to CFP^+^ ISCs and progenitor cells (Fig. S1B). Therefore, an increase in EEPs does not seem to be to the detriment of enteroblast formation. This result also suggests that the observed increase in (Pros^+^, GFP^+^) EEPs could potentially account for the non-significant higher percentage of GFP^+^ cells seen after 10 days of knockdown (Fig. 1B,C). Taken together, our results show that Rab21 loss in ISCs and progenitor cells increases the number of EEPs and mature enteroendocrine cells without affecting the number of enteroblasts. Next, we validated the specificity of *Rab21* knockdown on the enlarged enteroendocrine cell population by expressing an untagged and degenerated Rab21 construct that was not targeted by *Rab21* RNAis (Nassari et al., 2022). Notably, co-expression of degenerated Rab21 with two different *Rab21* RNAi constructs for 10 days in ISC and progenitor cells rescued the increased proportion of (GFP^−^, Pros^+^) mature enteroendocrine cells, as well as (GFP^+^, Pros^+^) EEPs. (Fig. 1D-F).

To explore the unique role of Rab21 in ISCs, we expressed both *Rab21* RNAis specifically in adult ISCs using an ISC-Gal4^ts^ driver line [Esg-Gal4, UAS-2xYFP; Su(H)-GBE-Gal80, tubGal80^ts^]. A constitutive Gal80 inhibits Gal4 activity in enteroblasts, resulting in transgene expression exclusively in ISCs (Wang et al., 2014; Zeng and Hou, 2015). ISC-specific Rab21 depletion for 10 days did not affect ISC proliferation, as revealed by the mitotic marker phospho-histone 3 (pH3) (Fig. 1G,H) and the percentage of total (YFP^+^, Pros^−^) ISCs (Fig. 1G,I). Still, the percentages of (YFP^+^, Pros^+^) EEPs and (YFP^−^, Pros^+^) mature enteroendocrine cells were significantly higher upon *Rab21* knockdown in ISCs (Fig. 1G,J,K). These results suggest that Rab21 in adult ISCs modulates enteroendocrine cell proportion.

### Basal autophagy inhibition in ISCs augments the mature enteroendocrine cell population

**Fig. 2.**
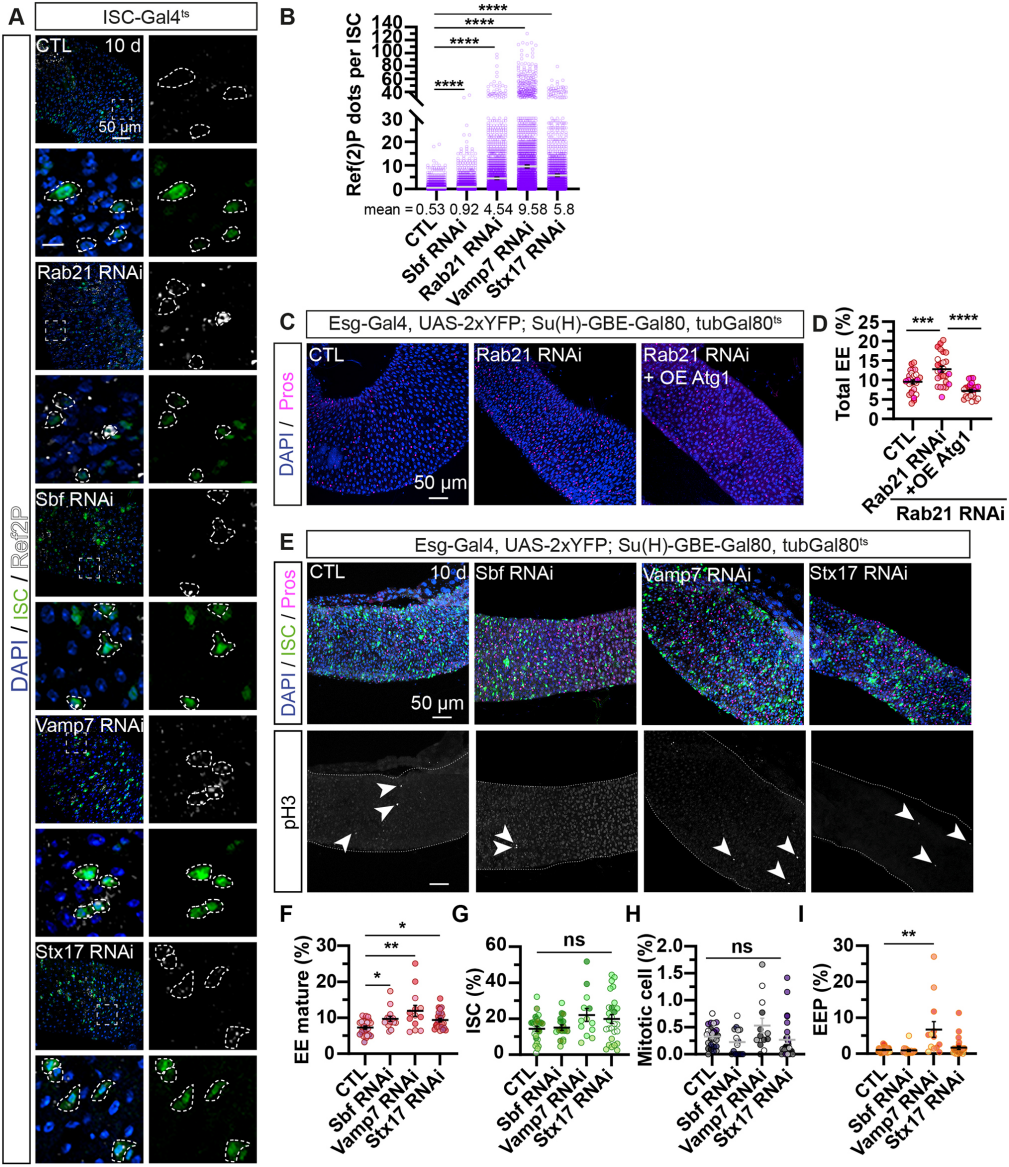
**Basal autophagy inhibition in intestinal stem cells augments the mature enteroendocrine cell population.** (A,B) Adult *Drosophila* posterior midgut from *esg-Gal4*, *UAS-2xYFP*; *Su(H)-GBE-Gal80*, *tubGal80^ts^* driver expressing *UAS-LacZ* (CTL), *Rab21* RNAi 1, *Sbf* RNAi 2, *Vamp7* RNAi or *Stx17* RNAi for 10 days, in ISCs. (A) Representative maximal projections. YFP labels ISCs (green), anti-Ref(2)P antibody marks autophagosome cargos (white), and DAPI stains nuclei. Scale bars: 50 μm; 10 μm (magnifications of boxed areas). Dashed lines outline GFP^+^ cells. (B) Quantification of Ref(2)P dots per YFP^+^ ISC, *n*≥10 guts and *n*≥1662 cells. (C,D) Adult *Drosophila* posterior midgut from *esg-Gal4*, *UAS-2xYFP*; *Su(H)-GBE-Gal80*, *tubGal80^ts^* driver expressing *UAS-LacZ* (CTL) and co-expressing *Rab21* RNAi 2 with *UAS-LacZ* or *UAS-Atg1* for 10 days, in ISCs. (C) Representative maximal projections. Anti-Pros antibody marks EE cells (magenta), and DAPI stains nuclei. Scale bar: 50 μm. (D) Quantification of the percentage of total Pros^+^ EE cells over total cells (DAPI^+^), *n*≥22 guts. (E-I) Adult *Drosophila* posterior midgut from *esg-Gal4*, *UAS-2xYFP*; *Su(H)-GBE-Gal80*, *tubGal80^ts^* driver expressing *UAS-LacZ* (CTL), *Rab21* RNAi 1, *Sbf* RNAi 2, *Vamp7* RNAi or *Stx17* RNAi for 10 days, in ISCs. (E) Representative maximal projections. YFP labels ISCs (green), anti-Pros antibody marks EEs (magenta), pH3 labels the mitotic ISCs, and DAPI stains nuclei. Scale bars: 50 μm. Arrowheads indicate pH3^+^ cells. (F-I) Quantification of the percentage of YFP^−^, Pros^+^ mature EEs over total cells (DAPI^+^) (F); YFP^+^, Pros^−^ ISCs over total cells (DAPI^+^) (G); pH3^+^ mitotic cells over total cells (DAPI^+^) (H); and YFP^+^, Pros^+^ EEPs over total cells (DAPI^+^) (I); *n*≥13 guts. *N*=three independent experiments from three independent crosses. Quantifications represent the mean±s.e.m. Each dot represents an intestine. (B) Each dot represents Ref(2)P puncta per cell. Kruskal–Wallis test was used, followed by Dunn's comparison tests. *P<0.05, **P<0.01, ***P<0.001, ****P<0.0001; ns, non-significant (*P*>0.05). Also see Figs S2 and S3.

Rab21 plays a role in autophagosome–lysosome fusion by regulating Vamp7 trafficking (Jean et al., 2015). To confirm that Rab21 loss decreases autophagy in ISCs, we quantified the number of autophagy cargo adaptors Ref(2)P (SQSTM1/p62 in mammals) per ISCs (Fig. 2A), as previously described in mammals and *Drosophila* (Devorkin and Gorski, 2014; Klionsky et al., 2016), by using a validated anti-Ref(2)P antibody (Wang et al., 2023a) (Fig. S1C,D). ISC-Gal4^ts^-driven knockdown of *Rab21*, *Vamp7* and *Stx17* led to a strong Ref(2)P accumulation within YFP^+^ ISCs, whereas upon Sbf depletion, Ref(2)P dots accumulated to a lower degree (Fig. 2A,B). These results correlate with a defect in the autophagy process as previously reported in various cell types following Rab21 depletion (Jean et al., 2015; Lauzier et al., 2019; Nassari et al., 2022; Dey et al., 2015). To assess whether the increase in enteroendocrine cells resulted from reduced autophagy upon *Rab21* knockdown in adult ISCs, we artificially enhanced autophagy in Rab21-depleted ISCs by overexpressing Atg1. Increasing Atg1 protein levels is sufficient to stimulate autophagosome formation and autophagosome–lysosome fusion (Bjedov et al., 2020; Juricic et al., 2022; Scott et al., 2007; Kim et al., 2011; Wang et al., 2018, 2023b). Notably, this rescued the increase in enteroendocrine cells caused by ISC-targeted Rab21 loss (Fig. 2C,D). Additionally, as Rab21 also regulates the WASP and SCAR homolog (WASH) complex and the sorting of various cargo (Pellinen et al., 2006; Del Olmo et al., 2019; Yang et al., 2012; Pei et al., 2022), we tested whether the loss of WASH complex subunits would phenocopy Rab21 loss. Further supporting a direct link among Rab21, autophagy and enteroendocrine cell formation, except for *CCDC53*, the knockdown of ISCs positive for *Wash*, *Strump* or *FAM21*, which encode subunits of the Wash complex, did not affect the number of enteroendocrine cells (Fig. S2A).

Using ISC-Gal4^ts^, inhibition of autophagosome–lysosome fusion by expressing RNAi against *Sbf Vamp7* or *Stx17* in ISCs for 10 days phenocopied the increase in the (Pros^+^, YFP^−^) mature enteroendocrine cell population (Fig. 2E,F), as observed in Rab21-depleted ISCs within the R5 region of intestines (Fig. 1G,J). Moreover, a higher percentage of the total number of enteroendocrine cells upon Sbf depletion was confirmed by using a different RNAi (Fig. S2B). Similarly to Rab21 loss in ISCs, knockdown of *Sbf*, *Vamp7* or *Stx17* did not affect the percentage of pH3^+^ mitotic cells (Fig. 2E,H) or of (YFP^+^, Pros^−^) ISCs. Nevertheless, we noticed that the population of (YFP^+^, Pros^−^) ISCs tended to increase upon Vamp7 or Stx17 depletion, but without reaching statistical significance (Fig. 2E,G). Finally, although the effect on (YFP^+^ and Pros^+^) EEPs was more subtle upon *Sbf* or *Stx17* knockdown, Vamp7 depletion in ISCs led to a significant increase in the percentage of EEPs (Fig. 2E,I). Altogether, these results suggest that limiting autophagosome–lysosome fusion in adult ISCs increases the number of mature enteroendocrine cells.

### Under homeostatic conditions, inhibition of basal autophagy promotes differentiation into enteroendocrine cells

**Fig. 3.**
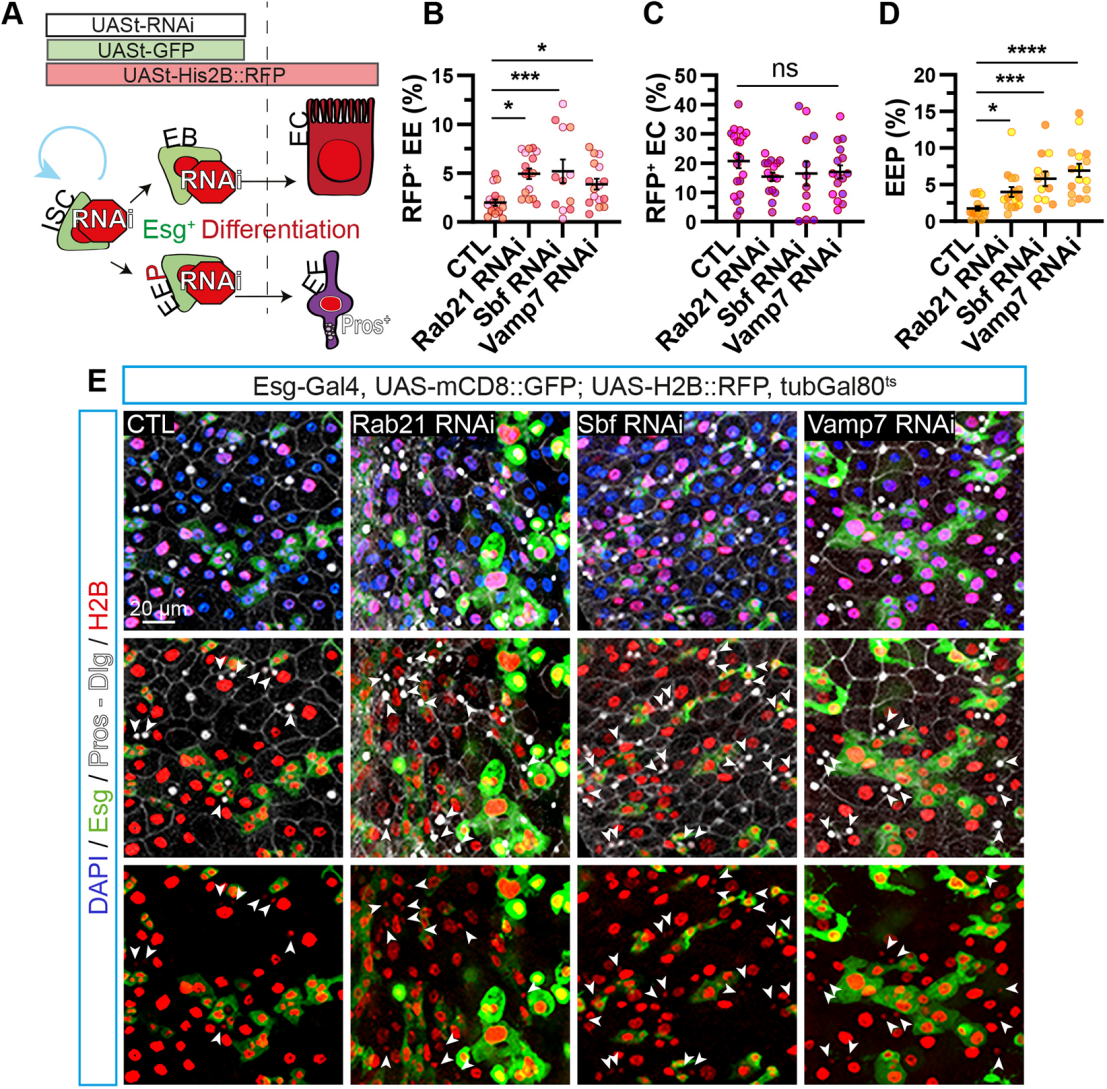
**Under homeostatic conditions, inhibiting basal autophagy favors differentiation into enteroendocrine cells.** (A) Schematic representation of the repressible dual differential stability cell marker (ReDDM) lineage-tracing method. (B-E) Adult *Drosophila* posterior midgut from *esg-Gal4*, *UAS-mCD8::GFP*; *UAS-H2B::RFP*, *tubGal80^ts^* driver expressing *UAS-LacZ* (CTL), *Rab21* RNAi 1, *Sbf* RNAi 1 or *Vamp7* RNAi for 10 days, in ISCs and progenitors. (B-D) Quantification of the percentage of (GFP^−^, RFP^+^ Pros^+^) EEs differentiated from depleted ISCs and progenitors over total cells (DAPI^+^) (B); (GFP^−^, RFP^+^ Pros^−^) enterocytes differentiated from depleted ISCs and progenitors over total cells (DAPI^+^) (C); (GFP^+^, RFP^+^, Pros^+^) EEPs over total cells (DAPI^+^) (D); *n*≥12 guts. (E) Representative maximal projections from the top to the lumen of the intestine. GFP labels Esg^+^ ISCs and progenitors (green), anti-Pros and anti-Discs large (Dlg) antibodies mark EEs and cell plasma membranes, respectively (white), H2B labels cell lineages from depleted ISCs and progenitors (red), and DAPI stains nuclei. Arrowheads show RFP^+^ Pros^+^ EEs. Scale bar: 20 µm. *N*=three independent experiments from three independent crosses. Kruskal–Wallis test was used, followed by Dunn's comparison tests. *P<0.05, ***P<0.001, ****P<0.0001; ns, non-significant (*P*>0.05). Also see Fig. S3.

Autophagy deficiency seems to disturb only the enteroendocrine cell population. To establish that dysfunctional autophagy leads to differentiation into enteroendocrine cells, we employed the Esg-Gal4 repressible dual differential stability cell marker (ReDDM) lineage-tracing method (Antonello et al., 2015). This technique enables the longitudinal tracking of stable RFP-labeled histone H2B progeny originating from ISCs subjected to RNA interference against autophagy-related genes. Differentiated daughter cells mature into (Pros^+^, GFP^−^, RFP^+^) enteroendocrine cells or (Pros^−^, GFP^−^, RFP^+^) enterocytes (Fig. 3A). Thus, we evaluated the rate of differentiation into both enteroendocrine cells and enterocytes upon autophagy inhibition. The percentage of new RFP-labeled mature enteroendocrine cells, which derived from knocked-down ISCs and progenitors, significantly increased upon Rab21, Sbf and Vamp7 depletion (Fig. 3B,E, arrowheads). Simultaneously, the percentage of new enterocytes remained close to that of the controls (Fig. 3C,E). This result correlates with an equal ratio of enteroblasts compared to the number of CFP^+^ ISCs and progenitors upon the depletion of Sbf, Vamp7 (Fig. S3) or Rab21 (Fig. S1B), in ISCs and progenitors.

Finally, knockdown of *Rab21*, *Sbf* and *Vamp7* in ISCs and progenitor cells was correlated with a higher percentage of EEPs (GFP^+^, RFP^+^ and Pros^+^) (Fig. 3D,E). Contrary to this observation, depletion of Sbf or Stx17 exclusively in ISCs using the ISC-Gal4^ts^ driver did not significantly increase the EEP population (Fig. 2I). As other GEFs or SNARE can activate Rab21 or modulate autophagosome–lysosome fusion, we hypothesized that Sbf or Stx17 depletion in both ISCs and their progenitors affects differentiation over a longer period, which might explain the differing phenotypes compared to those resulting from ISC-restricted depletion.

Therefore, inhibition of basal autophagy seems to enhance the rate of enteroendocrine cell differentiation without affecting the enterocyte population.

### Rab21 affects enteroendocrine differentiation by activating Stat92E

**Fig. 4.**
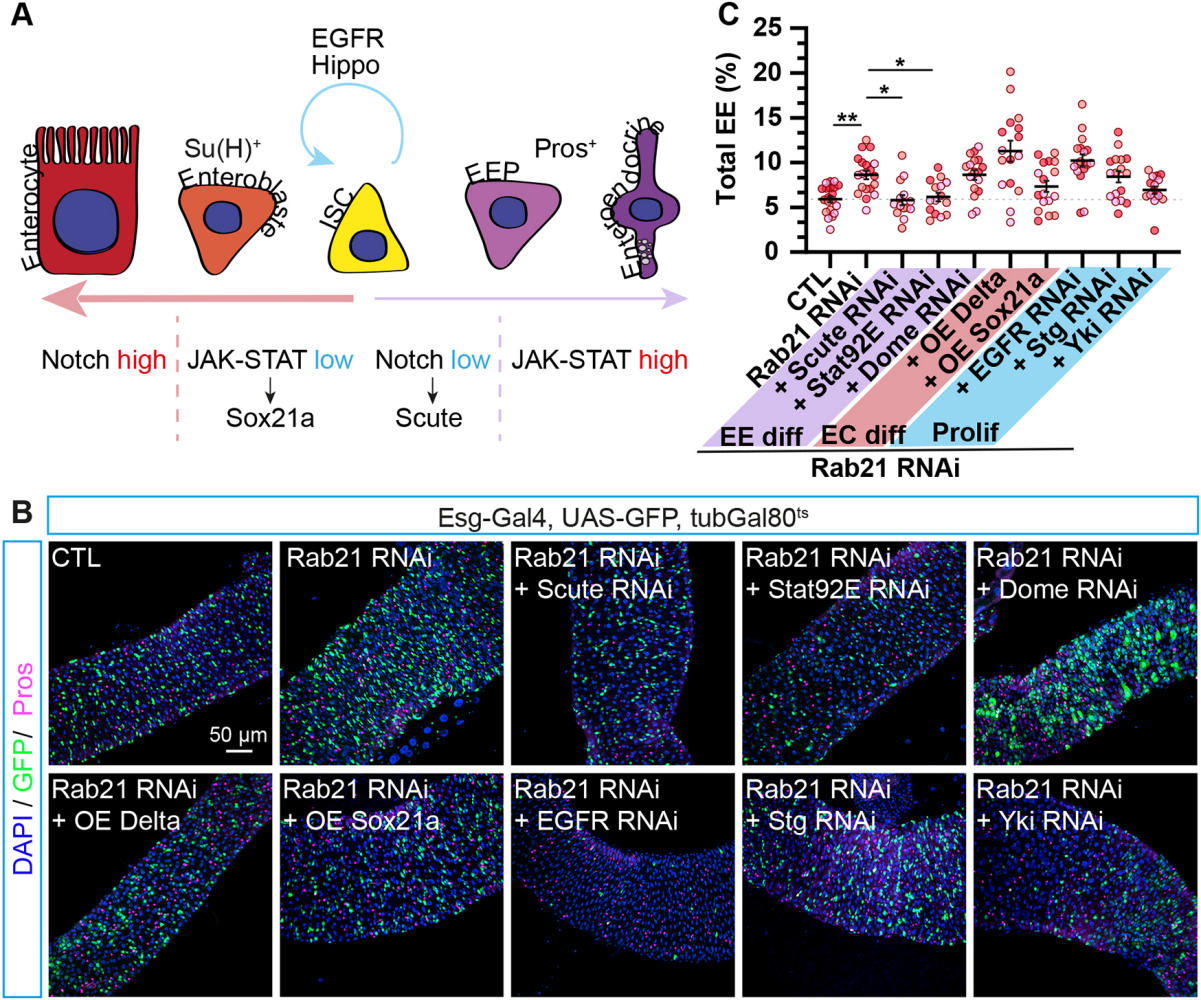
**Rescue experiments with cell fate modulators identify Stat92E as a Rab21 modifier for enteroendocrine cell differentiation.** (A) Schematic representation of signaling pathways regulating ISC lineages (Boumard and Bardin, 2021). The schematic was generated by C.L.-K. and is not a direct reproduction of previously published schematics. (B,C) Adult *Drosophila* posterior midgut from *esg-Gal4*, *UAS-GFP*, *TubGal80^ts^* driver expressing *UAS-LacZ* (CTL) or co-expressing *Rab21* RNAi 2 with *UAS-LacZ* or with cell fate modulators for 10 days in ISCs and progenitors. (B) Representative maximal projections. GFP labels ISCs and progenitor cells (green), anti-Pros antibody marks EEs (magenta), and DAPI stains nuclei. Scale bar: 50 µm. (C) Quantification of the percentage of total Pros^+^ EEs over total cells (DAPI^+^), *n*≥14 guts. Data information: *N*=three independent experiments from three independent crosses. Quantifications represent the mean±s.e.m. Each dot represents an intestine. The statistical tests used were one-way ANOVA followed by Dunnett's comparison tests. *P<0.05, **P<0.01; ns, non-significant (*P*>0.05).

We aimed to characterize the interplay between autophagy inhibition and pathways affecting enteroendocrine cell differentiation. We thus performed a targeted screen of known modulators of these processes in Rab21-depleted ISCs and progenitor cells (Esg-Gal4^ts^) (Fig. 4A). Enteroendocrine cell differentiation is inhibited by blocking, in the ISCs, the JAK-STAT pathway (Dome, Stat92E) or by depleting a transcriptional inducer of enteroendocrine differentiation (Scute) (Chen et al., 2018; Liu et al., 2010; Lin et al., 2010). By combining Rab21 loss with these RNAis, we found that Stat92E of the JAK-STAT pathway and Scute were potent modifiers (Fig. 4B,C).

Although we did not observe a statistically significant increase in ISCs and mitotic cells in the R5 region (Fig. 1) at 10 days, a previous study has shown that autophagy inhibition in ISCs augments their proliferation through EGFR activation (Zhang et al., 2019). To ensure that the observed increase in the percentage of enteroendocrine cells upon *Rab21* knockdown is not due to enhanced ISC proliferation before 10 days, we depleted three known regulators of ISC proliferation: *EGFR*, *String* and *Yorkie* (*Yki*) (Zhang et al., 2019; Ren et al., 2010). Notably, co-depletion of EGFR with Rab21 drastically reduced the number of GFP^+^ ISCs and progenitor cells, yet the proportion of enteroendocrine cells remained elevated (Fig. 4B,C). Similar results were observed with Stg and Yki depletion, although the effects on GFP^+^ cells were less pronounced. Taken together with our mitotic cell counts (Fig. 1), these findings suggest that, in the R5 region, the increase in enteroendocrine cells is most likely not a consequence of increased ISC proliferation.

Finally, we wanted to confirm that the higher number of enteroendocrine cells caused by Rab21 depletion is not an indirect consequence of decreased enterocyte differentiation. Delta overexpression in mosaic analysis with a repressible cell marker (MARCM) clones leads to clones completely formed by enterocytes, while upregulation of Sox21a can force enteroblast differentiation to enterocytes (Guo and Ohlstein, 2015; Zhai et al., 2017; Beebe et al., 2010). To further confirm that the population of enterocytes was not affected after 10 days of autophagy inhibition (as observed in Fig. 3), we promoted their differentiation by overexpressing Delta or Sox21a in the ISCs and their progenitors. Both Delta or Sox21a overexpression failed to rescue with statistical significance the increased percentage of enteroendocrine cells from Rab21 loss (Fig. 4B,C). In line with the lineage tracing method results (Fig. 3), this suggests that the higher number of enteroendocrine cells observed during Rab21 ISCs depletion was not an indirect consequence of fewer enterocytes. Taken together, this screen of cell fate modulators suggests an interplay between Rab21 and the JAK-STAT pathway to regulate enteroendocrine cell differentiation.

**Fig. 5.**
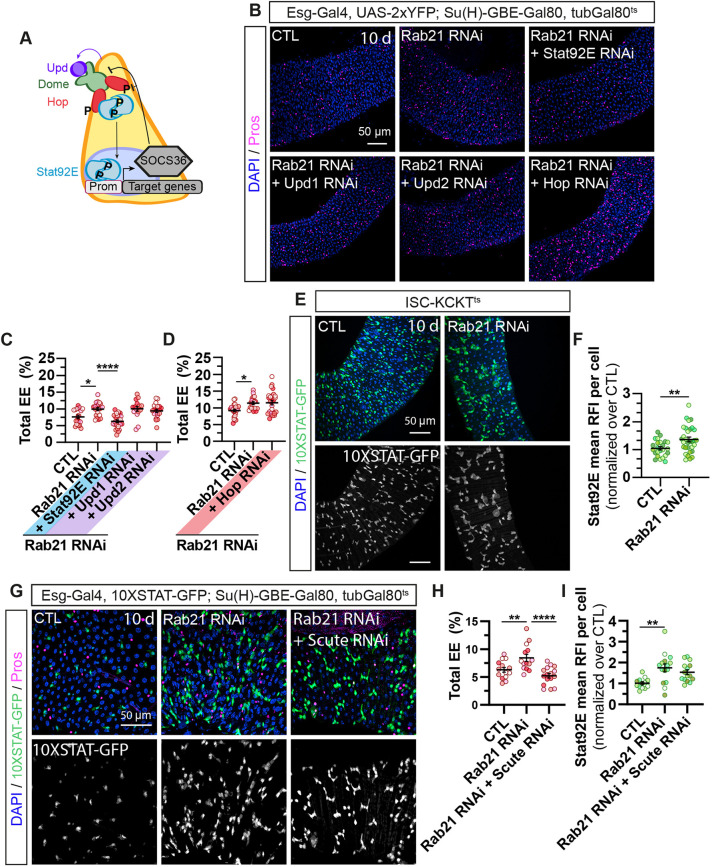
**Rab21 affects enteroendocrine differentiation by activating Stat92E downstream of Hop.** (A) Schematic representation of the JAK-STAT pathway (Herrera and Bach, 2019). The schematic was generated by C.L.-K. and is not a direct reproduction of previously published schematics. Dome, Domeless; Hop, Hopscotch; P, phosphorylation; Prom, promoter; SOCS36E, Suppressor of cytokine signaling 36E; Upd, Unpaired. (B-D) Adult *Drosophila* posterior midgut from *esg-Gal4*, *UAS-2xYFP*; *Su(H)-GBE-Gal80*, *tubGal80^ts^* driver expressing *UAS-GFP* (CTL) and co-expressing *Rab21* RNAi 2 with *UAS-LacZ* or *Stat92E* RNAi, *upd1* RNAi, *upd2* RNAi or *Hop* RNAi for 10 days, in ISCs. (B) Representative maximal projections. Anti-Pros antibody marks EEs (magenta), and DAPI stains nuclei. Scale bar: 50 µm. (C,D) Quantification of the percentage of total Pros^+^ EEs over total cells (DAPI^+^), *n*≥16 guts. (E,F) Adult *Drosophila* posterior midgut from ISC-KCKT^TS^ (ISC- intestinal-kickout-GAL4) driver co-expressing 10XSTAT-GFP reporter with *UAS-LacZ* (CTL) or *Rab21* RNAi 1. (E) Representative maximal projections. 10XSTAT-GFP labels cells with active Stat92E (green and white), and DAPI stains the nuclei. Scale bars: 50 µm. (F) Quantification of 10XSTAT-GFP mean relative fluorescence intensity (RFI) per cell per intestine normalized to the control, *n*≥27 guts. (G-I) Adult *Drosophila* posterior midgut from *esg-Gal4*, *10XSTAT-GFP*; *Su(H)-GBE-Gal80*, *tubGal80^ts^* driver expressing *UAS-mCD8:RFP* (CTL), or co-expressing *Rab21* RNAi 2 with *UAS-LacZ* or *scute* RNAi, for 10 days, in ISCs. (G) Representative maximal projections. 10XSTAT-GFP labels cells with active Stat92E (green and white). Anti-Pros antibody marks EEs (magenta), and DAPI stains nuclei. Scale bar: 50 µm. (H,I) Quantification of the percentage of total Pros^+^ EEs over total cells (DAPI^+^) (H) and the mean 10XSTAT-GFP RFI per cell normalized to the control per intestine (I); *n*≥15 guts and 1481 cells. *N*≥three independent experiments from at least three independent crosses. Quantifications represent the mean±s.e.m. Each dot represents the intestine. Statistical tests used were as follows: (F) Kruskal–Wallis test followed by Dunn's comparison test, (C,H,I) one-way ANOVA test followed by Dunnett's comparison test, and (D) unpaired one-tailed *t*-test. *P<0.05, **P<0.01, ****P<0.0001; ns, non-significant (*P*>0.05).

Therefore, we investigated the cell-autonomous effects of Rab21 in ISCs to understand how *Rab21* genetically interacts with the JAK-STAT pathway. The JAK-STAT pathway (Fig. 5A) is activated by the interaction between the membrane receptor Domeless (Dome) and secreted ligands Unpaired 1-3 (Upd1-3), leading to Hop kinase autophosphorylation (Herrera and Bach, 2019). Hop (JAK ortholog) phosphorylates Stat92E, which dimerizes and translocates to the nucleus to induce target gene expression, including the suppressor of the cytokine signaling family, to repress the pathway (Herrera and Bach, 2019). Three Upds activate the JAK-STAT pathway, with Upd1 and Upd2 secreted by ISCs and progenitor cells, and Upd3 secreted by enteroblasts and enterocytes (Herrera and Bach, 2019; Zhou et al., 2013). Because our focused screen targeted ISCs and progenitor cells (Esg-Gal4^ts^), we used the ISC-Gal4^ts^ driver and performed epistasis experiments, specifically in ISCs, to assess which JAK-STAT component(s) rescued Rab21 loss. Rab21 was co-depleted in ISCs using previously published RNAi constructs targeting *Hop*, *Stat92E*, *upd1* and *upd2* (Zhang et al., 2019; Chen et al., 2018; Rajan and Perrimon, 2012; Sanchez Bosch et al., 2019; Oliveira et al., 2023; Weber et al., 2003). Depletion of Upd1 and Upd2 did not restore the increased enteroendocrine cell percentage (Fig. 5B,C), which is in agreement with the lack of rescue by *dome* RNAi in the original screen (Fig. 4). Only *Stat92E* RNAi reduced the number of enteroendocrine cells, suggesting that Rab21 loss acts downstream of Hop to regulate enteroendocrine differentiation (Fig. 5B-D).

JAK-STAT signaling is important for progenitor differentiation into both enteroendocrine cells and enterocytes. Although Stat92E activation in enteroblasts is required for their differentiation into enterocytes via Sox21a expression (Zhai et al., 2017), hyperactivation of the JAK-STAT pathway in stem cells leads to an increased number of enteroendocrine cells (Lin et al., 2010). Therefore, we monitored Stat92E transcription activation using the 10XSTAT92E-GFP reporter, which correlates with GFP expression levels (Bach et al., 2007; Ekas et al., 2010). We confirmed that expressing a constitutively active form of Hop with the ISC-Gal4^ts^ driver is sufficient to enhance Stat92E activity and drastically increase the percentage of enteroendocrine cells (Fig. S4A-C).

We wondered whether Rab21 depletion in ISCs could increase JAK-STAT activity in these cells. Single-cell measurements of GFP relative fluorescence intensity revealed a statistically significant increase in Rab21-depleted ISCs compared to control ISCs. This was observed with two different *Rab21* RNAi constructs and in the context of two different ISC-specific drivers (Fig. 5E-I). Interestingly, co-expression of *Rab21* and *scute* RNAi specifically in ISCs rescued the increased percentage of enteroendocrine cells (Fig. 5G,H) but did not reduce the elevated Stat92E activity induced by Rab21 depletion in ISCs (Fig. 5G,I). This suggests that Scute counteracts Rab21 knockdown-induced enteroendocrine cell differentiation by acting downstream of Stat92E. Taken together, our data show that Rab21 inhibition in ISCs enhances Stat92E activity by acting downstream of Hop/JAK, thereby increasing enteroendocrine cell differentiation.

### Autophagy inhibition increases the enteroendocrine cell population through Stat92E activation

**Fig. 6.**
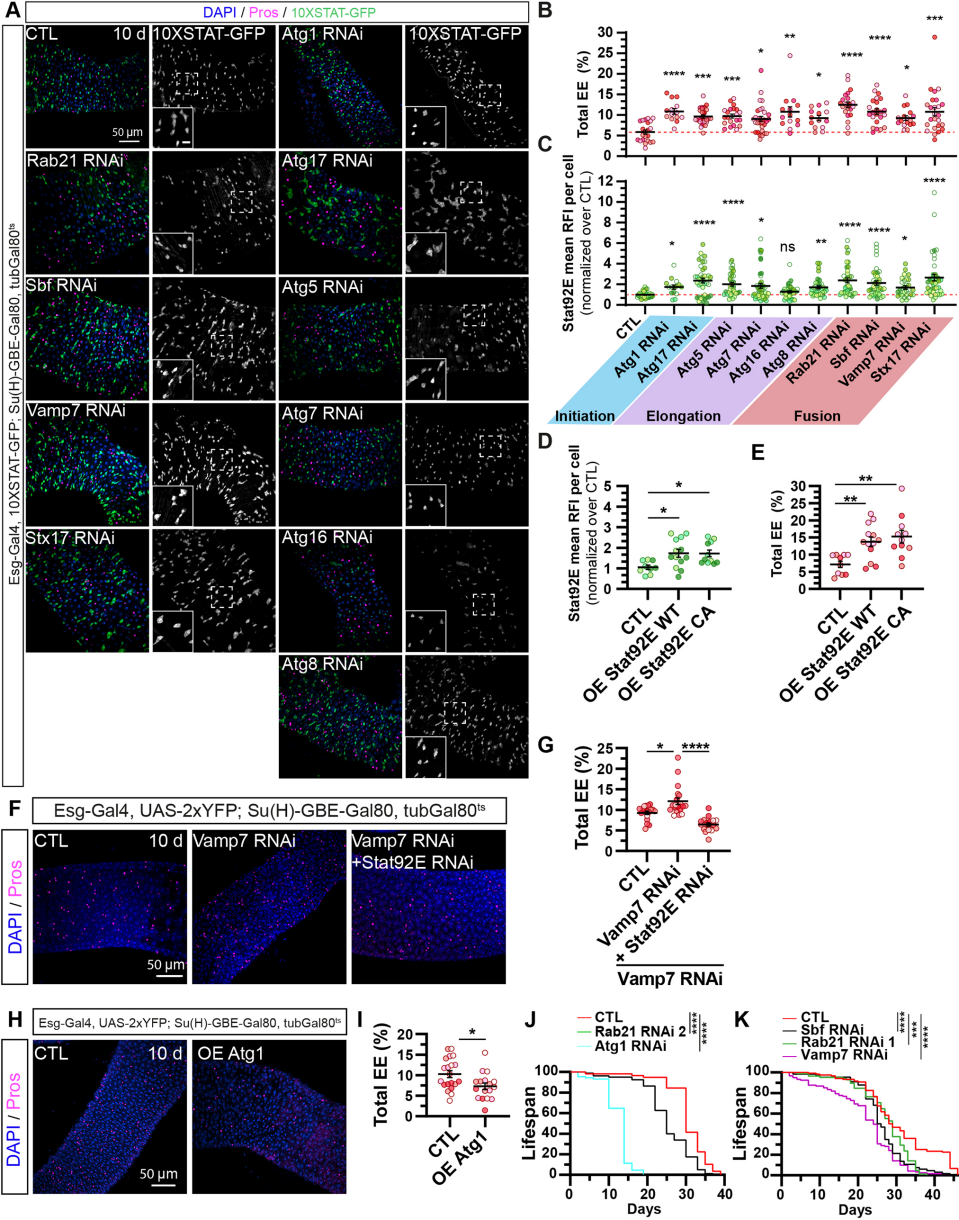
**Disrupting autophagy in ISCs increases the enteroendocrine cell population through Stat92E activation.** (A-E) Adult *Drosophila* posterior midgut from *esg-Gal4*, *10XSTAT-GFP*; *Su(H)-GBE-Gal80*, *tubGal80^ts^* driver expressing *UAS-mCD8:RFP* (CTL), RNAis against core Atg genes involved at different stages of autophagy, or overexpressing Stat92E wild type (WT) and Stat92E constitutive active (CA) for 10 days, in ISCs. (A) Representative maximal projections. 10XSTAT-GFP labels cells with active Stat92E (green and white), anti-Pros antibody marks EEs (magenta), and DAPI stains nuclei. Scale bars: 50 µm; 10 μm (insets). (B-E) Quantification of the percentage of total Pros^+^ EEs over total cells (DAPI^+^) (B,E) and mean 10XSTAT-GFP RFI per cell normalized to the control per intestine (C,D); *n*≥14 guts and 2525 cells. (F,G) Adult *Drosophila* posterior midgut from *esg-Gal4*, *UAS-2xYFP*; *Su(H)-GBE-Gal80*, *tubGal80^ts^* driver expressing *UAS-GFP* (CTL) and co-expressing *Vamp7* RNAi with *UAS-GFP* or *Stat92E* RNAi for 10 days, in ISCs. (F) Representative maximal projections. Anti-Pros antibody marks EEs (magenta), and DAPI stains nuclei. Scale bar: 50 µm. (G) Quantification of the percentage of total Pros^+^ EEs over total cells (DAPI^+^), *n*≥18 guts. (H,I) Adult *Drosophila* posterior midgut from *esg-Gal4*, *UAS-2xYFP*; *Su(H)-GBE-Gal80*, *tubGal80^ts^* driver expressing *UAS-LacZ* (CTL) or overexpressing Atg1 for 10 days in ISCs. (H) Representative maximal projections. Anti-Pros antibody marks EEs (magenta), and DAPI stains nuclei. Scale bar: 50 µm. (I) Quantification of the percentage of total Pros^+^ EEs over total cells (DAPI^+^), *n*≥17 guts. (J,K) Lifespan curves of female adult *Drosophila esg-Gal4*, *UAS-2xYFP*; *Su(H)-GBE-Gal80*, *tubGal80^ts^*, expressing *UAS-LacZ* (CTL) or RNAis against *Atg1*, *Rab21* (RNAi 1 and RNAi 2), *Sbf* or *Vamp7* in ISCs, *n*=66-121 female flies. *N*≥three independent experiments from at least three independent crosses. Quantifications represent the mean±s.e.m. Each dot represents an intestine. Statistical tests used were as follows: (B,C,G) Kruskal–Wallis test, followed by Dunn's comparison tests, (D,E,I) one-way ANOVA test followed by Dunnett's comparison test, and (J,K) Kaplan–Meier curves analyzed with a statistical log-rank Mantel-Cox test. *P<0.05, **P<0.01, ***P<0.001, ****P<0.0001; ns, non-significant (*P*>0.05). See also Figs S4 and S5.

Our results suggest that inhibiting autophagy regulators involved in autophagosome–lysosome fusion favors enteroendocrine cell differentiation. To extend these findings, we tested whether disrupting autophagosome formation or elongation would phenocopy Rab21, Sbf, Vamp7 and Stx17 loss by increasing the number of enteroendocrine cells and Stat92E activity. Using the ISC-Gal4^ts^ driver combined with the 10XSTAT92E-GFP reporter, RNAis against *Atg1* and *Atg17*, which control phagophore formation, and *Atg5*, *Atg7*, *Atg8* and *Atg16*, which control autophagosome elongation, were specifically expressed in ISCs (Fig. 6A,B). Because the 10XSTAT92E-GFP reporter labels ISCs and progenitors (Beebe et al., 2010), we validated the efficiency of RNAi on autophagy by quantifying the number of Ref(2)P dots in GFP^+^ undifferentiated cells. The inhibition of all *Atg* genes increased the number of Ref(2)P dots (Fig. S4D,E). Interestingly, an increase in the percentage of enteroendocrine cells along with higher Stat92E activity was monitored after knocking down all Atg genes in ISCs for 10 days, except for *Atg16* (Fig. 6A-C). These phenotypes were not observed, however, after 5 days of knockdown (Fig. S5A-C). *Atg16* genetically interacts in an autophagy-independent manner with the Slit-Robo2 pathway involved in enteroendocrine cell differentiation (Nagy et al., 2017). This may explain the Stat92E-independent increase in enteroendocrine cells following *Atg16* knockdown. Interestingly, pH3^+^ mitotic ISCs remained unchanged after 5 days of autophagy inhibition, along with the enteroendocrine cell population and Stat92E activity (Fig. S5A-C). These results reinforce the hypothesis that autophagy inhibition for 10 days in ISCs increases the rate of enteroendocrine cell differentiation, rather than promoting ISC proliferation locally in the R5 region.

In adult ISCs, constitutive activation of the kinase Hop/JAK for 10 days (used as a positive control) markedly increased both Stat92E activity and the number of Pros^+^ enteroendocrine cells (Fig. S4A-C). Interestingly, overexpression of a highly active Stat92E^ΔNΔC^ mutant (Ekas et al., 2010) (Stat92E.CA), or simple overexpression of wild-type (WT) Stat92E, phenocopied the increased percentage of enteroendocrine cells and elevated Stat92E activity, as observed upon autophagy inhibition (Fig. 6D,E). These findings suggest that autophagy modulates Stat92E activity in adult ISCs to prevent excessive differentiation into enteroendocrine cells. To test this possibility directly, we inhibited *Vamp7* along with *Stat92E*, specifically in ISCs, and quantified the percentage of enteroendocrine cells. Similarly to the Rab21 loss, Stat92E depletion rescued the increase in the proportion of enteroendocrine cells caused by Vamp7 depletion (Fig. 6F,G). In addition, inducing autophagy specifically in adult ISCs by overexpressing Atg1 reciprocally decreased the percentage of enteroendocrine cells (Fig. 6H,I). These results support the notion that, in ISCs, autophagy regulates the rate of enteroendocrine differentiation. Inhibition of autophagy genes, regulating either the initiation or fusion steps, specifically in adult ISCs significantly shortened *Drosophila* lifespan (Fig. 6J,K). Interestingly, these findings align with published data showing that Scute overexpression or depletion in ISCs and progenitor cells, which respectively increase or decrease enteroendocrine cell numbers, also reduced *Drosophila* lifespan (Amcheslavsky et al., 2014). These data also suggest that the increase in enteroendocrine cells under autophagy inhibition may not be restricted to the posterior midgut. As such, we observed a higher percentage of enteroendocrine cells in the anterior part of the midgut after Atg5 depletion in adult ISCs over 10 days (Fig. S5D,E). Finally, enteroendocrine cell subtypes (Guo et al., 2019b) were analyzed by quantitative PCR (qPCR) performed on whole intestines expressing small interfering RNAs against autophagy regulators in ISCs. The data suggested a twofold enrichment of *Allatostatin C* (*AstC*)*^+^* class I enteroendocrine cells, and potentially of (*AstA^+^; AstC^+^*) and (*AstC^+^; AstA^+^; CCHA1^+^*) subclusters as well (Fig. S5F).

From these experiments, we conclude that autophagy regulates Stat92E activation to affect enteroendocrine cell differentiation, at basal state, which can be counteracted by the transcription factor Scute.

## DISCUSSION

Dysfunctional autophagy is associated with IBD, which causes chronic inflammation to impair the composition and function of the intestinal epithelium (Graham and Xavier, 2020; Larabi et al., 2019; Haq et al., 2019; Telpaz and Bel, 2023). Although proper intestinal regeneration requires a balance between ISC proliferation and differentiation (Beumer and Clevers, 2021), few studies have investigated the role of autophagy specifically in ISCs. In addition to its essential role in mitigating stressors and its requirement for the degradation of proteins and organelles (Li et al., 2023), autophagy is emerging as a regulator of cell fate in various types of adult stem cells (Lacarrière-Keïta et al., 2022; Clarke and Simon, 2019; Jeong et al., 2020). By targeting multiple autophagy regulators in ISCs and combining lineage tracing with a targeted screen, we demonstrated that autophagy inhibition increased Stat92E activity, causing an increase in enteroendocrine cells in the adult fly intestine. Our data provide evidence for the role of basal autophagy in the differentiation of enteroendocrine cells in the *Drosophila* adult intestine.

### In adult ISCs, basal autophagy limits Stat92E activity to modulate enteroendocrine cell differentiation without affecting the enterocyte population

In ISCs, a 10-day depletion of core autophagy genes (*Atg1*, *Atg5*, *Atg7*, *Atg8/LC3* and *Atg17/FIP200*), as well as auxiliary regulators (*Sbf*, *Rab21*, *Vamp7* and *Stx17*), increased the percentage of enteroendocrine cells (Figs 1, 2 and 6). Under physiological conditions, the hyperactivation of JAK-STAT in ISCs converts ISCs into enteroendocrine cells (Lin et al., 2010). Using a 10XSTAT-GFP reporter, we showed that autophagy inhibition augmented Stat92E activity (Figs 5 and 6). Our previous study demonstrated that the loss of Rab21 in enterocytes increased the Yki-induced expression of Upd3. Secreted Upd3 upregulates JAK/STAT signaling and increases the number of enteroendocrine cells (Nassari et al., 2022). Herein, the depletion of the receptor Dome, and Upd1 and Upd2 ligands, and Yki in adult ISCs failed to restore the number of enteroendocrine cells in the intestine (Figs 4 and 5). These results suggest that autophagy acts downstream of Hop to restrict Stat92E transcriptional activity and enteroendocrine differentiation.

JAK-STAT signaling plays well-established roles in enteroblast-to-enterocyte differentiation (Zhai et al., 2017; Zhou et al., 2013; Jiang et al., 2009), and it is surprising that we did not observe defects in the enterocyte population (Fig. 3). Our findings are consistent with a previous report showing that JAK-STAT overexpression in ISCs increased the number of enteroendocrine cells but not enterocytes (Zhai et al., 2015; Chen et al., 2016). In enteroblasts, JAK-STAT activation induces Sox21a expression, which is required for enterocyte differentiation (Zhai et al., 2017; Chen et al., 2016). Notably, Sox21a also requires Notch activation to promote enterocyte fate. In our study, overexpression of Sox21a under *Rab21* knockdown in ISCs and progenitors failed to fully restore basal enteroendocrine cell levels (Fig. 4). Previous studies have shown that Sox21a overexpression in ISCs alone is insufficient to induce enterocyte differentiation (Zhai et al., 2015; Chen et al., 2016). We observed increased enteroendocrine cell differentiation when JAK-STAT overactivation was restricted to ISCs (Fig. 6; Fig. S4B). Moreover, Stat92E overexpression induced a moderate increase in enteroendocrine cells compared to constitutive Hop activation. These findings suggest that Stat92E activity in ISCs may be thresholded to regulate enteroendocrine cell differentiation, likely independently of Sox21a, which may explain the lack of effect on enteroblasts (Figs S1 and S3) or enterocytes (Fig. 3). Interestingly, upon Stat92E overactivation due to Rab21-mediated autophagy inhibition, *scute* knockdown was sufficient to rescue enteroendocrine cell levels (Fig. 5G-I) without affecting Stat92E activity. This indicates that Scute levels counteract enteroendocrine cell differentiation induced by Stat92E. Further studies are needed to understand how autophagy regulates Stat92E activity in ISCs.

Because overexpression of Stat92E in ISCs is sufficient to drive enteroendocrine cell differentiation (Fig. 6), we hypothesized that autophagy may directly target Stat92E for degradation. It has been reported that STAT5 can be degraded under basal conditions in T-cell lymphocytes (Zhou et al., 2021), and in mesenchymal cells to limit osteoblast differentiation (Dieudonne et al., 2013). Interestingly, during infection, orbiviruses induce autophagic degradation of STAT2 to replicate in cells (Avia et al., 2019). Because viruses and other pathogens often hijack existing pathways to survive (Davey et al., 2011), the role of autophagy in controlling STAT levels is plausible. Further supporting this hypothesis, STAT1, STAT2 and STAT4 contain LC3-interacting motifs (Jacomin et al., 2016). Stat92E also contains an Atg8-interacting motif related to the LC3-interacting motif WxxL in mammals (Wild et al., 2014). Through this sequence, Stat92E could be recruited to autophagosomes for degradation (Birgisdottir et al., 2013). Another hypothesis is that autophagy might degrade positive regulators of Stat92E activity. Phosphorylated STAT proteins can interact with transcriptional co-activators such as EP300/CREB-binding protein (p300/CBP), nuclear factor-kappa B (NF-κB) or PU.1/SPI-1 (Pesu et al., 2003; Stütz and Woisetschläger, 1999; Gewinner et al., 2004; Wojciak et al., 2009). To enhance STAT6 activity, p300/CBP is recruited by p100 (Välineva et al., 2005). Autophagic degradation of p100 induces non-canonical activation of NF-κB (Chen et al., 2020). In contrast, in hematopoietic progenitor cells, autophagy degrades PU.1/Spi-1 transcription factor to repress Th9 lymphocyte differentiation (Rivera Vargas et al., 2017). Based on these studies, it would be interesting to investigate whether autophagy degrades or inhibits Nejire, Relish or Ets98B, which are the *Drosophila* orthologs of p300/CBP, p100 and PU.1/SPI-1, respectively, to restrict Stat92E activity. JAK-STAT is required for cell differentiation, as *Stat92E* loss-of-function mutants show decreased enteroendocrine cell and enterocyte differentiation (Lin et al., 2010; Beebe et al., 2010). However, this is independent of Upd activation because no impact on ISC proliferation or differentiation is observed in the null Upd mutant *Df(1)os1A* (Lin et al., 2010). This suggests the essential regulation of the JAK-STAT pathway under basal conditions. Our data revealed the intrinsic activation of Stat92E in ISCs and its modulation by autophagy to regulate enteroendocrine cell differentiation.

### Autophagy participates in the control of stem cell behavior

Previous studies have reported differences in stem cell behavior in response to dysfunctional autophagy. A decrease in proliferation (Nagy et al., 2018) associated with a loss of ISC number (Asano et al., 2017; Trentesaux et al., 2020; Nagy et al., 2018) has been reported in flies depleted of autophagy genes in ISCs and progenitors and in *Atg5* or *Atg7* KO mice in all epithelial cells. In contrast, in other contexts, autophagy inhibition has been shown to increase ISC proliferation both cell-autonomously and non-cell-autonomously via EGFR and Yki activation, respectively (Zhang et al., 2019; Zeng et al., 2015). These studies assessed proliferation throughout the entire intestine. However, stem cells can regionally replenish the intestinal epithelium by regulating local cell density and population dynamics (Marianes and Spradling, 2013; Buchon and Osman, 2015). Our study focused on the R5 region of the intestine, where Rab21 is highly expressed (Mattila et al., 2024). In ISCs from this region, where we observed an increase in the enteroendocrine population, knockdown of *Rab21*, *Sbf*, *Vamp7* or *Stx17* for 5 or 10 days did not significantly affect ISC proliferation, as indicated by pH3 labeling (Figs 1 and 2; Fig. S5). Furthermore, consistent with these findings, restricting ISC proliferation by knocking down *EGFR*, *Stg* or *Yki* did not reduce the percentage of enteroendocrine cells caused by Rab21 loss (Fig. 4). These results suggest that, in this region, the increase in enteroendocrine differentiation is likely independent of ISC proliferation.

Moreover, EEPs undergo an additional round of proliferation before expressing Pros, which represses their proliferation and triggers their final differentiation into enteroendocrine cells (Chen et al., 2018). This mechanism is mediated by Scute. Therefore, the extra round of EEP proliferation could account for the increased number of enteroendocrine cells without affecting ISC numbers. Co-depletion of Rab21 with Scute (Fig. 4) rescued the increase in enteroendocrine cells induced by Rab21 depletion, suggesting that autophagy inhibition may increase enteroendocrine cell numbers through this pathway, while leaving other cell types (ISCs, enteroblasts and enterocytes) unaffected at 10 days post-RNAi induction in the R5 region. Despite the observed higher percentage of enteroendocrine cells upon the depletion of Atg5 in ISCs within the anterior midgut, more studies will be required to extend our findings to the entire gut and to understand the function of autophagy in proliferation versus that in differentiation.

### Dysfunctional enteroendocrine cells are associated with IBD

Our study established a role for autophagy in ISC fate decisions toward enteroendocrine cell differentiation in the unstressed intestinal epithelium. As pathogenic infections can increase JAK-STAT signaling to drive enteroendocrine cell differentiation in flies (Liu et al., 2022), it will be of interest to test whether autophagy affects Stat92E activity following infection. Because infection-dependent activity requires ISC proliferation and depends on Dome and Hop (Liu et al., 2022), autophagy could differentially regulate Stat92E activity based on the cellular context. Therefore, understanding how autophagy regulates the ISC fate in response to environmental changes is a significant new research direction for future studies. In mammals, *Stat5* and *Stat3* KOs in all intestinal epithelial cells are detrimental to intestinal crypt regeneration, whereas the addition of interleukins improves intestinal crypt regeneration without being necessary for organoid generation (Gilbert et al., 2015; Lindemans et al., 2015). However, little is known about the role of STAT transcription factors family in ISCs. Translating our results to mammals could help us understand the regulation and function of JAK-STAT in the autophagy-defective mammalian intestinal epithelium. Autophagy and JAK-STAT components, as well as Phox2B enteroendocrine cell regulator, have been identified as IBD susceptibility genes (Rioux et al., 2007; Jostins et al., 2012). An increase in enteroendocrine cells has been reported in patients with Crohn's disease (Moran et al., 2012) as well as dysregulation of hormonal peptide secretion, which might correlate with dysfunctional transit in patients with IBD (Worthington et al., 2018). Although this result requires further confirmation, repressing autophagy for 10 days in adult ISCs appears to increase some subsets of enteroendocrine cells expressing *AstC* (Fig. S5F), which is involved in feeding behavior and glycemia regulation (Kubrak et al., 2022). Similar to flies, infections in mice can lead to an increase in enteroendocrine cell numbers and subtypes (Wang et al., 2007). As defective autophagy increases susceptibility to infections, our data suggest that the rise in enteroendocrine cells may represent an adaptive response by the organism to mitigate infectious risk. Importantly, as previously shown (Amcheslavsky et al., 2014), elevated levels of enteroendocrine cells shorten *Drosophila* lifespan (Fig. 6). Therefore, investigating whether this interplay between autophagy, JAK-STAT signaling and enteroendocrine cell fate is conserved in the mammalian intestine could help uncover altered mechanisms involved in pathogenic conditions such as IBD, especially given the growing use of JAK inhibitors in patients with IBD (Herrera-deGuise et al., 2023).

### Limitations of the study

Except for the qPCR analysis and Fig. S5D,E, all experiments were conducted in the posterior part of the posterior midgut. Further studies are needed to investigate whether autophagy inhibition in ISCs across different midgut regions could differentially affect Stat92E-mediated enteroendocrine cell differentiation. Moreover, although our data strongly suggest a relationship between autophagy and enteroendocrine cell differentiation, we cannot rule out a minor effect on enterocytes, as we were unable to detect them using the anti-Pdm1 antibody. Finally, exploratory qPCR analysis of hormonal peptides expression from whole guts provided a broad indication of a potential effect on enteroendocrine cell subsets. Unbiased RNA transcriptomics from sorted Pros^+^ enteroendocrine cells could enhance sensitivity and help characterize differential differentiation into enteroendocrine cell subsets following autophagy inhibition in adult ISCs.

## MATERIALS AND METHODS

### Reagents and resources

Table S1 provides details on all reagents and resources used in this study, including sources.

### Drosophila

The *Drosophila* stocks were conserved at room temperature (RT). Stocks and experimental flies were raised on a standard diet composed of 7 g/l agar, 60 g/l cornmeal, 60 g/l molasses, 23.5 g/l yeast extract, 4.5 ml/l Tegosept (BioShop) and 4 ml/l propionic acid.

### TARGET system

Cell-specific Gal4 lines were combined with a ubiquitously expressed thermosensitive Gal80, referred to as Gal4^ts^, to repress Gal4 transcriptional activity at a permissive temperature (18-20°C). Experimental crosses were maintained at 20°C to inhibit Gal4 activity until eclosion. F1 female flies were collected 1-3 days after eclosion and shifted to 29°C to alleviate Gal80 repression for 5 or 10 days (see figure legends). Every 2 days, flies were flipped onto fresh food.

### Lifespan

To collect enough progenies, 40-50 virgins were mated with 13-17 males in jars at 20°C and then removed 3 days after. Progenies were collected on the day of eclosion until the next day. For each genotype, several vials of ten to 15 adult females with four males were transferred at 29°C to express RNAis in ISCs. Dead or censored female flies were monitored every 2 days after being transferred into a new vial. ‘Censored’ refers to flies that either escaped or died during handling and were therefore excluded from the analysis.

### Intestine dissection and immunocytochemistry

The intestines from adult female flies were dissected in phosphate-buffered saline (PBS), fixed with 4% paraformaldehyde for 2 h at RT and transferred to PBS. To clean the lumen, the intestines were trimmed anteriorly and posteriorly to the posterior midgut, incubated in glycerol 50% in PBS for 20 min, and washed three times for 10 min with PBS-0.1% Triton X-100 at RT. After overnight incubation at 4°C with the primary antibodies diluted in PBS-0.1% Triton X-100, the intestines were washed three times for 10 min at RT with PBS-0.1% Triton X-100. Secondary antibody incubation was performed for at least 3 h at RT in PBS-0.1% Triton X-100, followed by three 10-min incubations with PBS-0.1% Triton X-100 and three 10-min incubations with PBS at RT. Nuclei were stained with DAPI (1:10,000) diluted in PBS for 20 min and rinsed twice with PBS for 10 min at RT. The intestines were incubated for 10-20 min in 50% glycerol PBS before being mounted in SlowFade Gold Antifade Mountant with or without DAPI. The following primary and secondary antibodies were used: mouse anti-Prosp (Developmental Studies Hybridoma Bank, 1:1000), rabbit anti-pH3 (Millipore, 1:1000), rabbit anti-Ref(2)P (Abcam, 1:1000), mouse anti-Discs large (1:250), Alexa anti-mouse 647 (Invitrogen, 1:1000) and Alexa anti-rabbit 546 (Invitrogen, 1:1000). See Table S1 for full details on antibodies used.

### Image acquisition

The posterior midgut R5 regions were imaged on a Zeiss LSM880 confocal microscope using either a 20× Plan-APOCHROMAT/0.8 numerical aperture (NA) or a 40× oil Plan-APOCHROMAT/1.4 NA objective. *Z*-stacks were performed from one side to the other of the midgut with 2 μm steps between images. For intensity measurement using the 10XSTAT-GFP activity reporter, *z*-stacks were acquired from one side until the lumen part with 1 μm steps between images. Representative images are maximal projections unless specified otherwise. The image levels were adjusted uniformly and linearly for all conditions within the same experiment using Photoshop software.

### Image analysis

For each intestine, all images were analyzed using an automated approach. *Z*-stack images were analyzed in 3D using IMARIS software to quantify intestinal cell populations. First, images were processed to diminish the background and to smooth the signal by applying a Gaussian filter of 0.6 μm and a background subtraction of 10 μm, 5 μm and 20 μm for nuclei (DAPI), Pros^+^ cells, and YFP^+^ or GFP^+^ Esg^+^ cells, respectively. Second, the objects were detected using spot or surface modules. Spots with a 3.5-4 μm *xy* diameter and a 5 μm *z* diameter identified DAPI and Pros^+^ cells. Surfaces were created to identify YFP^+^ or Esg^+^ cells expressing GFP. Third, the ‘Find spots close to surface’ module was used with a 0.5 or 1 μm threshold to identify co-labeled YFP^+^ or Esg^+^ cells with Pros to calculate the number of EEPs. This module distinguishes Pros ‘spots close to the surface’ representing EEPs (Pros^+^ and YFP^+^ or Esg^+^ cells) from ‘spots far from the surface’ representing mature enteroendocrine cells (Pros^+^ and ISC^−^ or Esg^−^ cells). This method was used to determine DAPI spots close to the YFP surface representing all the YFP^+^ cells. The number of ISCs was then calculated by substracting the number of EEPs by the total numer of YFP cells. The total number of cells per imaged intestine was determined by the total number of DAPI^+^ nuclei.

For lineage-tracing (ReDDM) analysis, images were processed as above and by applying a Gaussian filter of 0.6 μm and a background subtraction of 20 μm for RFP^+^ cells. Spot objects were created to identify DAPI, RFP^+^, Pros^+^ cells and surface for Esg^+^ cells. ‘Find spots close to surface’ module was used with a 0.5 threshold to colocalize Esg^+^ with RFP^+^ nuclei. The new objects ‘RFP spots far from Esg surface’, representing newly differentiated cells (RFP^+^ and Esg^−^ cells), were then colocalized with Pros^+^ or DAPI^+^ objects by using the ‘Colocalized spots’ module with a threshold of 5 μm. The newly differentiated enteroendocrine (Esg^−^,Pros^+^, RFP^+^) or enterocyte (Esg^−^,Pros^−^, RFP^+^) cells were identified. The total number of cells per imaged intestine was determined by the total number of DAPI nuclei.

Ref(2)P dots and YFP^+^ ISCs were identified by creating 0.7 μm spot objects and surface objects respectively. Then, the number of Ref(2)P dots per ISC was quantified using the ‘split spot into surface’ module.

For enteroblast quantification, CFP^+^ Esg^+^ cells and Su(H)-GFP^+^ enteroblasts were identified by creating spot objects with a 3.8 μm *xy* diameter and a 5 μm *z* diameter. The proportion of enteroblasts was obtained by dividing the number of Su(H)-GFP^+^ cells by the number of CFP^+^ Esg^+^ cells.

All percentage quantifications were calculated as the ratio of a specific cell type over the total number of cells within the imaged area, as done by others (Okumura et al., 2016; Bardin et al., 2010; Loza-Coll et al., 2014; Biteau and Jasper, 2014; Ho et al., 2022).

Measurement of 10XSTAT-GFP intensity was performed using CellProfiler. Maximal projections were generated using ImageJ software. First, the background was reduced using ‘rescale intensity’ and ‘reduce noise’ modules. Second, 10XSTAT-GFP^+^ cells were delimited from the processed images using ‘Identify primary object’. Finally, the ‘Measure object intensity’ module quantified the integrated GFP intensity from the unmodified images within the 10XSTAT-GFP^+^ cells’ primary object. For each experiment, the average intensity per cell per intestine was calculated and normalized to the average intensity of controls.

### RNA extraction and qPCR analysis

After 10 days of RNAi, the intestines were dissected and directly flash frozen. RNA was extracted from the whole gut using Trizol reagent, following the manufacturer's recommendations. After DNase treatment, cDNAs obtained from 500 ng of reverse-transcribed RNA using the Maxima First Strand cDNA Synthesis kit was analyzed by qPCR using Luna MasterMix. Primers (Table S2) previously used to describe and validate enteroendocrine cell subclusters, were used (Guo et al., 2019b) after validation of their efficiency with standard curves. qPCRs were performed using the CFX Opus 96 (Bio-Rad).

### Statistical analyses

All experiments were performed at least three times from three independent crosses, except for Fig. S5E,F. The number of dots in each graph represents individual guts or cells, and the minimal number of intestines quantified per panel is mentioned in the figure legends. The different shades and colors within a panel depict the data from a biological repeat. Statistical analyses were performed using GraphPad Prism software. Normality was first assessed, followed by a parametric or nonparametric test, depending on the outcome of the normality test. An unpaired one-tailed *t*-test or Mann–Whitney test was performed if only two conditions were compared. For multiple comparisons, one-way analysis of variance (ANOVA) or Kruskal–Wallis tests were performed, followed by Dunnett's or Dunn's tests. Lifespan data were represented with Kaplan–Meier curves by plotting for each dead or censored individual flies. The Mantel–Cox log-rank test was performed to statistically analyze the survival curves. *P*<0.05 was considered significant. Graphs representing data show means±s.e.m.

## Supplementary Material

10.1242/dmm.052214_sup1 Supplementary information

## References

[DMM052214C1] Amcheslavsky, A., Song, W., Li, Q., Nie, Y., Bragatto, I., Ferrandon, D., Perrimon, N. and Ip, Y. T. (2014). Enteroendocrine cells support intestinal stem-cell-mediated homeostasis in Drosophila. *Cell Rep.* 9, 32-39. 10.1016/j.celrep.2014.08.052 25263551 PMC4198943

[DMM052214C2] Antonello, Z. A., Reiff, T., Ballesta-Illan, E. and Dominguez, M. (2015). Robust intestinal homeostasis relies on cellular plasticity in enteroblasts mediated by miR-8-Escargot switch. *EMBO J.* 34, 2025-2041. 10.15252/embj.201591517 26077448 PMC4551350

[DMM052214C3] Ariyapala, I. S., Holsopple, J. M., Popodi, E. M., Hartwick, D. G., Kahsai, L., Cook, K. R. and Sokol, N. S. (2020). Identification of split-GAL4 drivers and enhancers that allow regional cell type manipulations of the Drosophila melanogaster intestine. *Genetics* 216, 891-903. 10.1534/genetics.120.303625 32988987 PMC7768249

[DMM052214C4] Asano, J., Sato, T., Ichinose, S., Kajita, M., Onai, N., Shimizu, S. and Ohteki, T. (2017). Intrinsic autophagy is required for the maintenance of intestinal stem cells and for irradiation-induced intestinal regeneration. *Cell Rep.* 20, 1050-1060. 10.1016/j.celrep.2017.07.019 28768191

[DMM052214C5] Avia, M., Rojas, J. M., Miorin, L., Pascual, E., Van Rijn, P. A., Martín, V., García-Sastre, A. and Sevilla, N. (2019). Virus-induced autophagic degradation of STAT2 as a mechanism for interferon signaling blockade. *EMBO Rep.* 20, e48766. 10.15252/embr.201948766 31603272 PMC6831997

[DMM052214C6] Bach, E. A., Ekas, L. A., Ayala-Camargo, A., Flaherty, M. S., Lee, H., Perrimon, N. and Baeg, G.-H. (2007). GFP reporters detect the activation of the Drosophila JAK/STAT pathway in vivo. *Gene Expr. Patterns* 7, 323-331. 10.1016/j.modgep.2006.08.003 17008134

[DMM052214C7] Bardin, A. J., Perdigoto, C. N., Southall, T. D., Brand, A. H. and Schweisguth, F. (2010). Transcriptional control of stem cell maintenance in the Drosophila intestine. *Development* 137, 705-714. 10.1242/dev.039404 20147375 PMC2827683

[DMM052214C8] Barrett, J. C., Hansoul, S., Nicolae, D. L., Cho, J. H., Duerr, R. H., Rioux, J. D., Brant, S. R., Silverberg, M. S., Taylor, K. D., Barmada, M. M. et al. (2008). Genome-wide association defines more than 30 distinct susceptibility loci for Crohn's disease. *Nat. Genet.* 40, 955-962. 10.1038/ng.175 18587394 PMC2574810

[DMM052214C9] Bas, L., Papinski, D., Licheva, M., Torggler, R., Rohringer, S., Schuschnig, M. and Kraft, C. (2018). Reconstitution reveals Ykt6 as the autophagosomal SNARE in autophagosome--vacuole fusion. *J. Cell Biol.* 217, 3656-3669. 10.1083/jcb.201804028 30097514 PMC6168255

[DMM052214C10] Beebe, K., Lee, W.-C. and Micchelli, C. A. (2010). JAK/STAT signaling coordinates stem cell proliferation and multilineage differentiation in the Drosophila intestinal stem cell lineage. *Dev. Biol.* 338, 28-37. 10.1016/j.ydbio.2009.10.045 19896937

[DMM052214C11] Bel, S., Pendse, M., Wang, Y., Li, Y., Ruhn, K. A., Hassell, B., Leal, T., Winter, S. E., Xavier, R. J. and Hooper, L. V. (2017). Paneth cells secrete lysozyme via secretory autophagy during bacterial infection of the intestine. *Science* 357, 1047-1052. 10.1126/science.aal4677 28751470 PMC5702267

[DMM052214C12] Beumer, J. and Clevers, H. (2021). Cell fate specification and differentiation in the adult mammalian intestine. *Nat. Rev. Mol. Cell Biol.* 22, 39-53. 10.1038/s41580-020-0278-0 32958874

[DMM052214C13] Birgisdottir, Å. B. and Johansen, T. (2020). Autophagy and endocytosis – interconnections and interdependencies. *J. Cell Sci.* 133, jcs228114. 10.1242/jcs.228114 32501285

[DMM052214C14] Birgisdottir, Å. B., Lamark, T. and Johansen, T. (2013). The LIR motif - crucial for selective autophagy. *J. Cell Sci.* 126, 3237-3247. 10.1242/jcs.126128 23908376

[DMM052214C15] Biteau, B. B. and Jasper, H. (2014). Slit/Robo signaling regulates cell fate decisions in the intestinal stem cell lineage of Drosophila. *Cell Rep.* 7, 1867-1875. 10.1016/j.celrep.2014.05.024 24931602 PMC4086754

[DMM052214C16] Bjedov, I., Cochemé, H. M., Foley, A., Wieser, D., Woodling, N. S., Castillo-Quan, J. I., Norvaisas, P., Lujan, C., Regan, J. C., Toivonen, J. M. et al. (2020). Fine-tuning autophagy maximises lifespan and is associated with changes in mitochondrial gene expression in Drosophila. *PLoS Genet.* 16, 1-33. 10.1371/journal.pgen.1009083 PMC7738165 33253201

[DMM052214C17] Boumard, B. and Bardin, A. J. (2021). An amuse-bouche of stem cell regulation: Underlying principles and mechanisms from adult Drosophila intestinal stem cells. *Curr. Opin. Cell Biol.* 73, 58-68. 10.1016/j.ceb.2021.05.007 34217969

[DMM052214C18] Buchon, N. and Osman, D. (2015). All for one and one for all: Regionalization of the Drosophila intestine. *Insect Biochem. Mol. Biol.* 67, 2-8. 10.1016/j.ibmb.2015.05.015 26044368

[DMM052214C19] Cadwell, K., Liu, J. Y., Brown, S. L., Miyoshi, H., Loh, J., Lennerz, J. K., Kishi, C., Kc, W., Carrero, J. A., Hunt, S. et al. (2008). A key role for autophagy and the autophagy gene Atg16l1 in mouse and human intestinal Paneth cells. *Nature* 456, 259-263. 10.1038/nature07416 18849966 PMC2695978

[DMM052214C20] Capo, F., Wilson, A. and Di Cara, F. (2019). The intestine of Drosophila melanogaster: An emerging versatile model system to study intestinal epithelial homeostasis and host-microbial interactions in humans. *Microorganisms* 7, 336. 10.3390/microorganisms7090336 31505811 PMC6780840

[DMM052214C21] Chen, J., Xu, N., Huang, H., Cai, T. and Xi, R. (2016). A feedback amplification loop between stem cells and their progeny promotes tissue regeneration and tumorigenesis. *eLife* 5, e14330. 10.7554/eLife.14330 27187149 PMC4905741

[DMM052214C22] Chen, J., Xu, N., Wang, C., Huang, P., Huang, H., Jin, Z., Yu, Z., Cai, T., Jiao, R. and Xi, R. (2018). Author Correction: Transient Scute activation via a self-stimulatory loop directs enteroendocrine cell pair specification from self-renewing intestinal stem cells. *Nat. Cell Biol.* 20, 991-991. 10.1038/s41556-018-0053-z 29674680

[DMM052214C23] Chen, M., Zhao, Z., Meng, Q., Liang, P., Su, Z., Wu, Y., Huang, J. and Cui, J. (2020). TRIM14 promotes noncanonical NF-κB activation by modulating p100/p52 stability via selective autophagy. *Adv. Sci.* 7, 1901261. 10.1002/advs.201901261 PMC6947505 31921549

[DMM052214C24] Clarke, A. J. and Simon, A. K. (2019). Autophagy in the renewal, differentiation and homeostasis of immune cells. *Nat. Rev. Immunol.* 19, 170-183. 10.1038/s41577-018-0095-2 30531943

[DMM052214C25] Cornick, S., Kumar, M., Moreau, F., Gaisano, H. and Chadee, K. (2019). VAMP8-mediated MUC2 mucin exocytosis from colonic goblet cells maintains innate intestinal homeostasis. *Nat. Commun.* 10, 4306. 10.1038/s41467-019-11811-8 31541089 PMC6754373

[DMM052214C26] Davey, N. E., Travé, G. and Gibson, T. J. (2011). How viruses hijack cell regulation. *Trends Biochem. Sci.* 36, 159-169. 10.1016/j.tibs.2010.10.002 21146412

[DMM052214C27] Del Olmo, T., Lacarrière-Keïta, C., Normandin, C., Jean, D., Boisvert, F.-M. and Jean, S. (2019). RAB21 interacts with TMED10 and modulates its localization and abundance. *Biol. Open* 8, bio045336. 10.1242/bio.045336 31455601 PMC6777364

[DMM052214C28] del Valle Rodríguez, A., Didiano, D. and Desplan, C. (2011). Power tools for gene expression and clonal analysis in Drosophila. *Nat. Methods* 9, 47-55. 10.1038/nmeth.1800 22205518 PMC3574576

[DMM052214C29] DeVorkin, L. and Gorski, S. M. (2014). Monitoring autophagic flux using ref(2)P, the Drosophila p62 ortholog. *Cold Spring Harb. Protoc.* 2014, 959-966. 10.1101/pdb.prot080333 25183816

[DMM052214C30] Dey, G., Jaimovich, A., Collins, S. R., Seki, A. and Meyer, T. (2015). Systematic discovery of human gene function and principles of modular organization through phylogenetic profiling. *Cell Rep.* 10, 993-1006. 10.1016/j.celrep.2015.01.025 25683721 PMC5016211

[DMM052214C31] Dieudonne, F.-X., Sévère, N., Biosse-Duplan, M., Weng, J.-J., Su, Y. and Marie, P. J. (2013). Promotion of osteoblast differentiation in mesenchymal cells through Cbl-mediated control of STAT5 activity. *Stem Cells* 31, 1340-1349. 10.1002/stem.1380 23533197

[DMM052214C32] Ekas, L. A., Cardozo, T. J., Flaherty, M. S., McMillan, E. A., Gonsalves, F. C. and Bach, E. A. (2010). Characterization of a dominant-active STAT that promotes tumorigenesis in Drosophila. *Dev. Biol.* 344, 621-636. 10.1016/j.ydbio.2010.05.497 20501334 PMC2914209

[DMM052214C33] Franke, A., McGovern, D. P. B., Barrett, J. C., Wang, K., Radford-Smith, G. L., Ahmad, T., Lees, C. W., Balschun, T., Lee, J., Roberts, R. et al. (2010). Genome-wide meta-analysis increases to 71 the number of confirmed Crohn's disease susceptibility loci. *Nat. Genet.* 42, 1118-1125. 10.1038/ng.717 21102463 PMC3299551

[DMM052214C34] Funk, M. C., Zhou, J. and Boutros, M. (2020). Ageing, metabolism and the intestine. *EMBO Rep.* 21, e50047. 10.15252/embr.202050047 32567155 PMC7332987

[DMM052214C35] Galluzzi, L., Yamazaki, T. and Kroemer, G. (2018). Linking cellular stress responses to systemic homeostasis. *Nat. Rev. Mol. Cell Biol.* 19, 731-745. 10.1038/s41580-018-0068-0 30305710

[DMM052214C36] Gao, J., Reggiori, F. and Ungermann, C. (2018). A novel in vitro assay reveals SNARE topology and the role of Ykt6 in autophagosome fusion with vacuoles. *J. Cell Biol.* 217, 3670-3682. 10.1083/jcb.201804039 30097515 PMC6168247

[DMM052214C37] Gewinner, C., Hart, G., Zachara, N., Cole, R., Beisenherz-Huss, C. and Groner, B. (2004). The coactivator of transcription CREB-binding protein interacts preferentially with the glycosylated form of Stat5. *J. Biol. Chem.* 279, 3563-3572. 10.1074/jbc.M306449200 14597631

[DMM052214C38] Gilbert, S., Nivarthi, H., Mayhew, C. N., Lo, Y.-H., Noah, T. K., Vallance, J., Rülicke, T., Müller, M., Jegga, A. G., Tang, W. et al. (2015). Activated STAT5 confers resistance to intestinal injury by increasing intestinal stem cell proliferation and regeneration. *Stem Cell Rep.* 4, 209-225. 10.1016/j.stemcr.2014.12.004 PMC4325270 25579133

[DMM052214C39] Graham, D. B. and Xavier, R. J. (2020). Pathway paradigms revealed from the genetics of inflammatory bowel disease. *Nature* 578, 527-539. 10.1038/s41586-020-2025-2 32103191 PMC7871366

[DMM052214C40] Gumeni, S., Papanagnou, E.-D., Manola, M. S. and Trougakos, I. P. (2021). Nrf2 activation induces mitophagy and reverses Parkin/Pink1 knock down-mediated neuronal and muscle degeneration phenotypes. *Cell Death Dis.* 12, 671. 10.1038/s41419-021-03952-w 34218254 PMC8254809

[DMM052214C41] Guo, Z. and Ohlstein, B. (2015). Bidirectional Notch signaling regulates Drosophila intestinal stem cell multipotency. *Science* 350, aab0988. 10.1126/science.aab0988 26586765 PMC5431284

[DMM052214C42] Guo, T., Nan, Z., Miao, C., Jin, X., Yang, W., Wang, Z., Tu, Y., Bao, H., Lyu, J., Zheng, H. et al. (2019a). The autophagy-related gene Atg101 in Drosophila regulates both neuron and midgut homeostasis. *J. Biol. Chem.* 294, 5666-5676. 10.1074/jbc.RA118.006069 30760524 PMC6462509

[DMM052214C43] Guo, X., Yin, C., Yang, F., Zhang, Y., Huang, H., Wang, J., Deng, B., Cai, T., Rao, Y. and Xi, R. (2019b). The cellular diversity and transcription factor code of Drosophila enteroendocrine cells. *Cell Rep.* 29, 4172-4185.e5. 10.1016/j.celrep.2019.11.048 31851941

[DMM052214C44] Haq, S., Grondin, J., Banskota, S. and Khan, W. I. (2019). Autophagy: roles in intestinal mucosal homeostasis and inflammation. *J. Biomed. Sci.* 26, 19. 10.1186/s12929-019-0512-2 30764829 PMC6375151

[DMM052214C45] Harrison, D. A., Binari, R., Nahreini, T. S., Gilman, M. and Perrimon, N. (1995). Activation of a Drosophila Janus kinase (JAK) causes hematopoietic neoplasia and developmental defects. *EMBO J.* 14, 2857-2865. 10.1002/j.1460-2075.1995.tb07285.x 7796812 PMC398404

[DMM052214C46] Henckaerts, L., Cleynen, I., Brinar, M., John, J. M., Van Steen, K., Rutgeerts, P. and Vermeire, S. (2011). Genetic variation in the autophagy gene ULK1 and risk of Crohn's disease. *Inflamm. Bowel Dis.* 17, 1392-1397. 10.1002/ibd.21486 21560199

[DMM052214C47] Herrera, S. C. and Bach, E. A. (2019). JAK/STAT signaling in stem cells and regeneration: from Drosophila to vertebrates. *Development* 146, dev167643. 10.1242/dev.167643 30696713 PMC6361132

[DMM052214C48] Herrera-deGuise, C., Serra-Ruiz, X., Lastiri, E. and Borruel, N. (2023). JAK inhibitors: a new dawn for oral therapies in inflammatory bowel diseases. *Front. Med.* 10, 1089099. 10.3389/fmed.2023.1089099 PMC10017532 36936239

[DMM052214C49] Ho, M. T., Lu, J., Vazquez-Pianzola, P. and Suter, B. (2022). α-Phenylalanyl tRNA synthetase competes with Notch signaling through its N-terminal domain. *PLoS Genet.* 18, e1010185. 10.1371/journal.pgen.1010185 35486661 PMC9094542

[DMM052214C50] Itakura, E., Kishi-Itakura, C. and Mizushima, N. (2012). The hairpin-type tail-anchored SNARE Syntaxin 17 targets to autophagosomes for fusion with endosomes/lysosomes. *Cell* 151, 1256-1269. 10.1016/j.cell.2012.11.001 23217709

[DMM052214C51] Jacomin, A.-C., Samavedam, S., Promponas, V., Nezis, I. P., Jacomin, A.-C., Samavedam, S., Promponas, V. and Nezis, I. P. (2016). iLIR database: a web resource for LIR motif- containing proteins in eukaryotes. *Autophagy* 12, 1945-1953. 10.1080/15548627.2016.1207016 27484196 PMC5079668

[DMM052214C52] Jasper, H. (2020). Intestinal stem cell aging: origins and interventions. *Annu. Rev. Physiol.* 82, 203-226. 10.1146/annurev-physiol-021119-034359 31610128

[DMM052214C53] Jean, S., Cox, S., Nassari, S. and Kiger, A. A. (2015). Starvation-induced MTMR13 and RAB21 activity regulates VAMP8 to promote autophagosome-lysosome fusion. *EMBO Rep.* 16, 297-311. 10.15252/embr.201439464 25648148 PMC4364869

[DMM052214C54] Jeong, D., Qomaladewi, N. P., Lee, J., Park, S. H. and Cho, J. Y. (2020). The role of autophagy in skin fibroblasts, keratinocytes, melanocytes, and epidermal stem cells. *J. Invest. Dermatol.* 140, 1691-1697. 10.1016/j.jid.2019.11.023 32800183

[DMM052214C55] Jiang, H., Patel, P. H., Kohlmaier, A., Grenley, M. O., McEwen, D. G. and Edgar, B. A. (2009). Cytokine/Jak/Stat signaling mediates regeneration and homeostasis in the Drosophila midgut. *Cell* 137, 1343-1355. 10.1016/j.cell.2009.05.014 19563763 PMC2753793

[DMM052214C56] Jostins, L., Ripke, S., Weersma, R. K., Duerr, R. H., McGovern, D. P., Hui, K. Y., Lee, J. C., Schumm, L. P., Sharma, Y., Anderson, C. A. et al. (2012). Host-microbe interactions have shaped the genetic architecture of inflammatory bowel disease. *Nature* 491, 119-124. 10.1038/nature11582 23128233 PMC3491803

[DMM052214C57] Jung, H., Leal-Ekman, J. S., Lu, Q. and Stappenbeck, T. S. (2019). Atg14 protects the intestinal epithelium from TNF-triggered villus atrophy. *Autophagy* 15, 1990-2001. 10.1080/15548627.2019.1596495 30894050 PMC6844524

[DMM052214C58] Juricic, P., Lu, Y.-X., Leech, T., Drews, L. F., Paulitz, J., Lu, J., Nespital, T., Azami, S., Regan, J. C., Funk, E. et al. (2022). Long-lasting geroprotection from brief rapamycin treatment in early adulthood by persistently increased intestinal autophagy. *Nat. Aging* 2, 824-836. 10.1038/s43587-022-00278-w 37118497 PMC10154223

[DMM052214C59] Kim, J., Kundu, M., Viollet, B. and Guan, K.-L. (2011). AMPK and mTOR regulate autophagy through direct phosphorylation of Ulk1. *Nat. Cell Biol.* 13, 132-141. 10.1038/ncb2152 21258367 PMC3987946

[DMM052214C60] Kiral, F. R., Linneweber, G. A., Mathejczyk, T., Georgiev, S. V., Wernet, M. F., Hassan, B. A., von Kleist, M. and Hiesinger, P. R. (2020). Autophagy-dependent filopodial kinetics restrict synaptic partner choice during Drosophila brain wiring. *Nat. Commun.* 11, 1325. 10.1038/s41467-020-14781-4 32165611 PMC7067798

[DMM052214C61] Klionsky, D. J., Abdelmohsen, K., Abe, A., Abedin, M. J., Abeliovich, H., Acevedo Arozena, A., Adachi, H., Adams, C. M., Adams, P. D., Adeli, K. et al. (2016). Guidelines for use and interpretation of assays for monitoring autophagy (3rd edition). *Autophagy* 12, 1-222. 10.1080/15548627.2015.1100356 26799652 PMC4835977

[DMM052214C62] Korzelius, J., Naumann, S. K., Loza-Coll, M. A., Chan, J. S. K., Dutta, D., Oberheim, J., Gläßer, C., Southall, T. D., Brand, A. H., Jones, D. L. et al. (2014). Escargot maintains stemness and suppresses differentiation in Drosophila intestinal stem cells. *EMBO J.* 33, 2967-2982. 10.15252/embj.201489072 25298397 PMC4282643

[DMM052214C63] Kubrak, O., Koyama, T., Ahrentløv, N., Jensen, L., Malita, A., Naseem, M. T., Lassen, M., Nagy, S., Texada, M. J., Halberg, K. V. et al. (2022). The gut hormone Allatostatin C/Somatostatin regulates food intake and metabolic homeostasis under nutrient stress. *Nat. Commun.* 13, 692. 10.1038/s41467-022-28268-x 35121731 PMC8816919

[DMM052214C64] Lacarrière-Keïta, C., Nassari, S. and Jean, S. (2022). Autophagy in cell fate decisions: knowledge gained from Drosophila. *Genome* 65, 573-584. 10.1139/gen-2022-0069 36240515

[DMM052214C65] Larabi, A., Barnich, N. and Nguyen, H. T. T. (2019). New insights into the interplay between autophagy, gut microbiota and inflammatory responses in IBD. *Autophagy* 16, 38-51. 10.1080/15548627.2019.1635384 31286804 PMC6984609

[DMM052214C66] Lassen, K. G., Kuballa, P., Conway, K. L., Patel, K. K., Becker, C. E., Peloquin, J. M., Villablanca, E. J., Norman, J. M., Liu, T.-C., Heath, R. J. et al. (2014). Atg16L1 T300A variant decreases selective autophagy resulting in altered cytokine signaling and decreased antibacterial defense. *Proc. Natl Acad. Sci. USA* 111, 7741-7746. 10.1073/pnas.1407001111 24821797 PMC4040621

[DMM052214C67] Lauzier, A., Normandeau-Guimond, J., Vaillancourt-Lavigueur, V., Boivin, V., Charbonneau, M., Rivard, N., Scott, M. S., Dubois, C. M. and Jean, S. (2019). Colorectal cancer cells respond differentially to autophagy inhibition in vivo. *Sci. Rep.* 9, 11316. 10.1038/s41598-019-47659-7 31383875 PMC6683171

[DMM052214C68] Li, M., Tripathi-Giesgen, I., Schulman, B. A., Baumeister, W. and Wilfling, F. (2023). In situ snapshots along a mammalian selective autophagy pathway. *Proc. Natl. Acad. Sci. USA* 120, e2221712120. 10.1073/pnas.2221712120 36917659 PMC10041112

[DMM052214C69] Lin, G., Xu, N. and Xi, R. (2010). Paracrine unpaired signaling through the JAK/STAT pathway controls self-renewal and lineage differentiation of drosophila intestinal stem cells. *J. Mol. Cell Biol.* 2, 37-49. 10.1093/jmcb/mjp028 19797317

[DMM052214C70] Lindemans, C. A., Calafiore, M., Mertelsmann, A. M., O'Connor, M. H., Dudakov, J. A., Jenq, R. R., Velardi, E., Young, L. F., Smith, O. M., Lawrence, G. et al. (2015). Interleukin-22 promotes intestinal-stem-cell-mediated epithelial regeneration. *Nature* 528, 560-564. 10.1038/nature16460 26649819 PMC4720437

[DMM052214C71] Liu, W., Singh, S. R. and Hou, S. X. (2010). JAK-STAT is restrained by Notch to control cell proliferation of the Drosophila intestinal stem cells. *J. Cell. Biochem.* 109, 992-999. 10.1002/jcb.22482 20082318 PMC2893559

[DMM052214C72] Liu, X., Nagy, P., Bonfini, A., Houtz, P., Bing, X.-L., Yang, X. and Buchon, N. (2022). Microbes affect gut epithelial cell composition through immune-dependent regulation of intestinal stem cell differentiation. *Cell Rep.* 38, 110572. 10.1016/j.celrep.2022.110572 35354023 PMC9078081

[DMM052214C73] Lőrincz, P. and Juhász, G. (2019). Autophagosome-lysosome fusion. *J. Mol. Biol.* 432, 2462-2482. 10.1016/J.JMB.2019.10.028 31682838

[DMM052214C74] Loza-Coll, M. A., Southall, T. D., Sandall, S. L., Brand, A. H. and Jones, D. L. (2014). Regulation of Drosophila intestinal stem cell maintenance and differentiation by the transcription factor Escargot. *EMBO J.* 33, 2983-2996. 10.15252/embj.201489050 25433031 PMC4282644

[DMM052214C75] Marianes, A. and Spradling, A. C. (2013). Physiological and stem cell compartmentalization within the Drosophila midgut. *eLife* 2, e00886. 10.7554/eLife.00886 23991285 PMC3755342

[DMM052214C76] Matsui, T., Jiang, P., Nakano, S., Sakamaki, Y., Yamamoto, H. and Mizushima, N. (2018). Autophagosomal YKT6 is required for fusion with lysosomes independently of syntaxin 17. *J. Cell Biol.* 217, 2633-2645. 10.1083/jcb.201712058 29789439 PMC6080929

[DMM052214C77] Matsuzawa-Ishimoto, Y., Shono, Y., Gomez, L. E., Hubbard-Lucey, V. M., Cammer, M., Neil, J., Dewan, M. Z., Lieberman, S. R., Lazrak, A., Marinis, J. M. et al. (2017). Autophagy protein ATG16L1 prevents necroptosis in the intestinal epithelium. *J. Exp. Med.* 214, 3687-3705. 10.1084/jem.20170558 29089374 PMC5716041

[DMM052214C78] Mattila, J., Viitanen, A., Fabris, G., Strutynska, T., Korzelius, J. and Hietakangas, V. (2024). Stem cell mTOR signaling directs region-specific cell fate decisions during intestinal nutrient adaptation. *Sci. Adv.* 10, eadi2671. 10.1126/sciadv.adi2671 38335286 PMC10857434

[DMM052214C79] McGuire, S. E., Mao, Z. and Davis, R. L. (2004). Spatiotemporal gene expression targeting with the TARGET and gene-switch systems in Drosophila. *Sci. Signal.* 2004, pl6. 10.1126/stke.2202004pl6 14970377

[DMM052214C80] Micchelli, C. A. and Perrimon, N. (2006). Evidence that stem cells reside in the adult Drosophila midgut epithelium. *Nature* 439, 475-479. 10.1038/nature04371 16340959

[DMM052214C81] Moran, G. W., Pennock, J. and McLaughlin, J. T. (2012). Enteroendocrine cells in terminal ileal Crohn's disease. *J. Crohns. Colitis* 6, 871-880. 10.1016/j.crohns.2012.01.013 22398079

[DMM052214C82] Nagai, H., Tatara, H., Tanaka-Furuhashi, K., Kurata, S. and Yano, T. (2021). Homeostatic regulation of ROS-triggered Hippo-Yki pathway via autophagic clearance of Ref(2)P/p62 in the Drosophila intestine. *Dev. Cell* 56, 81-94.e10. 10.1016/j.devcel.2020.12.007 33400912

[DMM052214C83] Nagy, P., Szatmári, Z., Sándor, G. O., Lippai, M., Hegedűs, K. and Juhász, G. (2017). *Drosophila* Atg16 promotes enteroendocrine cell differentiation via regulation of intestinal Slit/Robo signaling. *Development* 144, 3990-4001. 10.1242/dev.147033 28982685

[DMM052214C84] Nagy, P., Sándor, G. O. and Juhász, G. (2018). Autophagy maintains stem cells and intestinal homeostasis in Drosophila. *Sci. Rep.* 8, 4644. 10.1038/s41598-018-23065-3 29545557 PMC5854693

[DMM052214C85] Nassari, S., Lacarrière-Keïta, C., Lévesque, D., Boisvert, F.-M. and Jean, S. (2022). Rab21 in enterocytes participates in intestinal epithelium maintenance. *Mol. Biol. Cell* 33, mbcE21030139. 10.1091/mbc.E21-03-0139 PMC9250356 35171715

[DMM052214C86] Nighot, P. K., Hu, C.-A. A. and Ma, T. Y. (2015). Autophagy enhances intestinal epithelial tight junction barrier function by targeting claudin-2 protein degradation. *J. Biol. Chem.* 290, 7234-7246. 10.1074/jbc.M114.597492 25616664 PMC4358142

[DMM052214C87] O'Brien, L. E., Soliman, S. S., Li, X. and Bilder, D. (2011). Altered modes of stem cell division drive adaptive intestinal growth. *Cell* 147, 603-614. 10.1016/j.cell.2011.08.048 22036568 PMC3246009

[DMM052214C88] Ohlstein, B. and Spradling, A. (2006). The adult Drosophila posterior midgut is maintained by pluripotent stem cells. *Nature* 439, 470-474. 10.1038/nature04333 16340960

[DMM052214C89] Okumura, T., Takeda, K., Kuchiki, M., Akaishi, M., Taniguchi, K. and Adachi-Yamada, T. (2016). GATAe regulates intestinal stem cell maintenance and differentiation in Drosophila adult midgut. *Dev. Biol.* 410, 24-35. 10.1016/j.ydbio.2015.12.017 26719127

[DMM052214C90] Oliveira, A. C., Santos, M., Pinho, M. and Lopes, C. S. (2023). String/Cdc25 phosphatase is a suppressor of Tau-associated neurodegeneration. *Dis. Model. Mech.* 16, dmm049693. 10.1242/dmm.049693 36601903 PMC9903143

[DMM052214C91] Patel, K. K., Miyoshi, H., Beatty, W. L., Head, R. D., Malvin, N. P., Cadwell, K., Guan, J.-L., Saitoh, T., Akira, S., Seglen, P. O. et al. (2013). Autophagy proteins control goblet cell function by potentiating reactive oxygen species production. *EMBO J.* 32, 3130-3144. 10.1038/emboj.2013.233 24185898 PMC3981139

[DMM052214C92] Pei, Y., Lv, S., Shi, Y., Jia, J., Ma, M., Han, H., Zhang, R., Tan, J. and Zhang, X. (2022). RAB21 controls autophagy and cellular energy homeostasis by regulating retromer-mediated recycling of SLC2A1/GLUT1. *Autophagy* 19, 1070-1086. 10.1080/15548627.2022.2114271 35993307 PMC10012929

[DMM052214C93] Pellinen, T., Arjonen, A., Vuoriluoto, K., Kallio, K., Fransen, J. A. M. and Ivaska, J. (2006). Small GTPase Rab21 regulates cell adhesion and controls endosomal traffic of β1-integrins. *J. Cell Biol.* 173, 767-780. 10.1083/jcb.200509019 16754960 PMC2063892

[DMM052214C94] Pesu, M., Aittomäki, S., Välineva, T. and Silvennoinen, O. (2003). PU.1 is required for transcriptional activation of the Stat6 response element in the Igepsilon promoter. *Eur. J. Immunol.* 33, 1727-1735. 10.1002/eji.200323680 12778491

[DMM052214C95] Rajan, A. and Perrimon, N. (2012). Drosophila cytokine unpaired 2 regulates physiological homeostasis by remotely controlling insulin secretion. *Cell* 151, 123-137. 10.1016/j.cell.2012.08.019 23021220 PMC3475207

[DMM052214C96] Ren, F., Wang, B., Yue, T., Yun, E.-Y., Ip, Y. T. and Jiang, J. (2010). Hippo signaling regulates Drosophila intestine stem cell proliferation through multiple pathways. *Proc. Natl Acad. Sci. USA* 107, 21064-21069. 10.1073/pnas.1012759107 21078993 PMC3000252

[DMM052214C97] Riffelmacher, T., Clarke, A., Richter, F. C., Stranks, A., Pandey, S., Danielli, S., Hublitz, P., Yu, Z., Johnson, E., Schwerd, T. et al. (2017). Autophagy-dependent generation of free fatty acids is critical for normal neutrophil differentiation. *Immunity* 47, 466-480.e5. 10.1016/j.immuni.2017.08.005 28916263 PMC5610174

[DMM052214C98] Rioux, J. D., Xavier, R. J., Taylor, K. D., Silverberg, M. S., Goyette, P., Huett, A., Green, T., Kuballa, P., Barmada, M. M., Datta, L. W. et al. (2007). Genome-wide association study identifies new susceptibility loci for Crohn disease and implicates autophagy in disease pathogenesis. *Nat. Genet.* 39, 596-604. 10.1038/ng2032 17435756 PMC2757939

[DMM052214C99] Rivera Vargas, T., Cai, Z., Shen, Y., Dosset, M., Benoit-Lizon, I., Martin, T., Roussey, A., Flavell, R. A., Ghiringhelli, F. and Apetoh, L. (2017). Selective degradation of PU.1 during autophagy represses the differentiation and antitumour activity of TH9 cells. *Nat. Commun.* 8, 559. 10.1038/s41467-017-00468-w 28916785 PMC5602674

[DMM052214C100] Sakai, Y., Takahashi, S., Koyama-Honda, I., Saito, C. and Mizushima, N. (2024). Experimental determination and mathematical modeling of standard shapes of forming autophagosomes. *Nat. Commun.* 15, 91. 10.1038/s41467-023-44442-1 38167876 PMC10762205

[DMM052214C101] Sanchez Bosch, P., Makhijani, K., Herboso, L., Gold, K. S., Baginsky, R., Woodcock, K. J., Alexander, B., Kukar, K., Corcoran, S., Jacobs, T. et al. (2019). Adult Drosophila lack hematopoiesis but rely on a blood cell reservoir at the respiratory epithelia to relay infection signals to surrounding tissues. *Dev. Cell* 51, 787-803.e5. 10.1016/j.devcel.2019.10.017 31735669 PMC7263735

[DMM052214C102] Scott, R. C., Juhász, G. and Neufeld, T. P. (2007). Direct induction of autophagy by Atg1 inhibits cell growth and induces apoptotic cell death. *Curr. Biol.* 17, 1-11. 10.1016/j.cub.2006.10.053 17208179 PMC1865528

[DMM052214C103] Sênos Demarco, R., Uyemura, B. S. and Jones, D. L. (2020). EGFR signaling stimulates autophagy to regulate stem cell maintenance and lipid homeostasis in the Drosophila testis. *Cell Rep.* 30, 1101-1116.e5. 10.1016/j.celrep.2019.12.086 31995752 PMC7357864

[DMM052214C104] Shatz, O. and Elazar, Z. (2024). The physiological relevance of autophagosome morphogenesis. *Trends Biochem. Sci.* 49, 569-572. 10.1016/j.tibs.2024.05.002 38796312

[DMM052214C105] Stütz, A. M. and Woisetschläger, M. (1999). Functional synergism of STAT6 with either NF-κB or PU.1 to mediate IL-4-induced activation of IgE germline gene transcription. *J. Immunol.* 163, 4383-4391. 10.4049/jimmunol.163.8.4383 10510379

[DMM052214C106] Takáts, S., Glatz, G., Szenci, G., Boda, A., Horváth, G. V., Hegedűs, K., Kovács, A. L. and Juhász, G. (2018). Non-canonical role of the SNARE protein Ykt6 in autophagosome-lysosome fusion. *PLoS Genet.* 14, e1007359. 10.1371/journal.pgen.1007359 29694367 PMC5937789

[DMM052214C107] Telpaz, S. and Bel, S. (2023). Autophagy in intestinal epithelial cells prevents gut inflammation. *Trends Cell Biol.* 33, 817-819. 10.1016/j.tcb.2023.07.010 37586983

[DMM052214C108] Trentesaux, C., Fraudeau, M., Pitasi, C. L., Lemarchand, J., Jacques, S., Duche, A., Letourneur, F., Naser, E., Bailly, K., Schmitt, A. et al. (2020). Essential role for autophagy protein ATG7 in the maintenance of intestinal stem cell integrity. *Proc. Natl. Acad. Sci. USA* 117, 11136-11146. 10.1073/pnas.1917174117 32371487 PMC7245075

[DMM052214C109] Ulgherait, M., Rana, A., Rera, M., Graniel, J. and Walker, D. W. (2014). AMPK modulates tissue and organismal aging in a non-cell-autonomous manner. *Cell Rep.* 8, 1767-1780. 10.1016/j.celrep.2014.08.006 25199830 PMC4177313

[DMM052214C110] Välineva, T., Yang, J., Palovuori, R. and Silvennoinen, O. (2005). The transcriptional co-activator protein p100 recruits histone acetyltransferase activity to STAT6 and mediates interaction between the CREB-binding protein and STAT6. *J. Biol. Chem.* 280, 14989-14996. 10.1074/jbc.M410465200 15695802

[DMM052214C111] Wang, H., Steeds, J., Motomura, Y., Deng, Y., Verma-Gandhu, M., El-Sharkawy, R. T., McLaughlin, J. T., Grencis, R. K. and Khan, W. I. (2007). CD4+ T cell-mediated immunological control of enterochromaffin cell hyperplasia and 5-hydroxytryptamine production in enteric infection. *Gut* 56, 949-957. 10.1136/gut.2006.103226 17303597 PMC1994360

[DMM052214C112] Wang, L., Zeng, X., Ryoo, H. D. and Jasper, H. (2014). Integration of UPRER and oxidative stress signaling in the control of intestinal stem cell proliferation. *PLoS Genet.* 10, e1004568. 10.1371/journal.pgen.1004568 25166757 PMC4148219

[DMM052214C113] Wang, C., Wang, H., Zhang, D., Luo, W., Liu, R., Xu, D., Diao, L., Liao, L. and Liu, Z. (2018). Phosphorylation of ULK1 affects autophagosome fusion and links chaperone-mediated autophagy to macroautophagy. *Nat. Commun.* 9, 3492. 10.1038/s41467-018-05449-1 30154410 PMC6113293

[DMM052214C114] Wang, H., Wang, X., Zhang, K., Wang, Q., Cao, X., Wang, Z., Zhang, S., Li, A., Liu, K. and Fang, Y. (2019). Rapid depletion of ESCRT protein Vps4 underlies injury-induced autophagic impediment and Wallerian degeneration. *Sci. Adv.* 5, eaav4971. 10.1126/sciadv.aav4971 30788439 PMC6374107

[DMM052214C115] Wang, R., Fortier, T. M., Chai, F., Miao, G., Shen, J. L., Restrepo, L. J., DiGiacomo, J. J., Velentzas, P. D. and Baehrecke, E. H. (2023a). PINK1, Keap1, and Rtnl1 regulate selective clearance of endoplasmic reticulum during development. *Cell* 186, 4172-4188.e18. 10.1016/j.cell.2023.08.008 37633267 PMC10530463

[DMM052214C116] Wang, Y., Que, H., Li, C. P., Wu, Z., Jian, F., Zhao, Y., Tang, H., Chen, Y., Gao, S., Wong, C. C. L. et al. (2023b). ULK phosphorylation of STX17 controls autophagosome maturation via FLNA. *J. Cell Biol.* 222, e202211025. 10.1083/jcb.202211025 37389864 PMC10316704

[DMM052214C117] Warr, M. R., Binnewies, M., Flach, J., Reynaud, D., Garg, T., Malhotra, R., Debnath, J. and Passegué, E. (2013). FOXO3A directs a protective autophagy program in haematopoietic stem cells. *Nature* 494, 323-327. 10.1038/nature11895 23389440 PMC3579002

[DMM052214C118] Weber, U. and Mlodzik, M. (2017). APC/CFzr/Cdh1-dependent regulation of planar cell polarity establishment via Nek2 kinase acting on Dishevelled. *Dev. Cell* 40, 53-66. 10.1016/j.devcel.2016.12.006 28041906 PMC5225046

[DMM052214C119] Weber, U., Eroglu, C. and Mlodzik, M. (2003). Phospholipid membrane composition affects EGF receptor and Notch signaling through effects on endocytosis during Drosophila development. *Dev. Cell* 5, 559-570. 10.1016/S1534-5807(03)00273-9 14536058

[DMM052214C120] Wild, P., McEwan, D. G. and Dikic, I. (2014). The LC3 interactome at a glance. *J. Cell Sci.* 127, 3-9. 10.1242/jcs.140426 24345374

[DMM052214C121] Wojciak, J. M., Martinez-Yamout, M. A., Dyson, H. J. and Wright, P. E. (2009). Structural basis for recruitment of CBP/p300 coactivators by STAT1 and STAT2 transactivation domains. *EMBO J.* 28, 948-958. 10.1038/emboj.2009.30 19214187 PMC2670858

[DMM052214C122] Worthington, J. J., Reimann, F. and Gribble, F. M. (2018). Enteroendocrine cells-sensory sentinels of the intestinal environment and orchestrators of mucosal immunity. *Mucosal Immunol.* 11, 3-20. 10.1038/mi.2017.73 28853441

[DMM052214C123] Yamamoto, H., Zhang, S. and Mizushima, N. (2023). Autophagy genes in biology and disease. *Nat. Rev. Genet.* 24, 382-400. 10.1038/s41576-022-00562-w 36635405 PMC9838376

[DMM052214C124] Yang, X., Zhang, Y., Li, S., Liu, C., Jin, Z., Wang, Y., Ren, F. and Chang, Z. (2012). Rab21 attenuates EGF-mediated MAPK signaling through enhancing EGFR internalization and degradation. *Biochem. Biophys. Res. Commun.* 421, 651-657. 10.1016/j.bbrc.2012.04.049 22525675

[DMM052214C125] Zeng, X. and Hou, S. X. (2015). Enteroendocrine cells are generated from stem cells through a distinct progenitor in the adult Drosophila posterior midgut. *Development* 142, 644-653. 10.1242/dev.113357 25670791 PMC4325374

[DMM052214C126] Zeng, X., Han, L., Singh, S. R., Liu, H., Neumüller, R. A., Yan, D., Hu, Y., Liu, Y., Liu, W., Lin, X. et al. (2015). Genome-wide RNAi screen identifies networks involved in intestinal stem cell regulation in Drosophila. *Cell Rep.* 10, 1226-1238. 10.1016/j.celrep.2015.01.051 25704823 PMC4420031

[DMM052214C127] Zhai, Z., Kondo, S., Ha, N., Boquete, J.-P., Brunner, M., Ueda, R. and Lemaitre, B. (2015). Accumulation of differentiating intestinal stem cell progenies drives tumorigenesis. *Nat. Commun.* 6, 10219. 10.1038/ncomms10219 26690827 PMC4703904

[DMM052214C128] Zhai, Z., Boquete, J.-P. and Lemaitre, B. (2017). A genetic framework controlling the differentiation of intestinal stem cells during regeneration in Drosophila. *PLoS Genet.* 13, e1006854. 10.1371/journal.pgen.1006854 28662029 PMC5510897

[DMM052214C129] Zhang, P., Holowatyj, A. N., Roy, T., Pronovost, S. M., Marchetti, M., Liu, H., Ulrich, C. M. and Edgar, B. A. (2019). An SH3PX1-dependent endocytosis-autophagy network restrains intestinal stem cell proliferation by counteracting EGFR-ERK signaling. *Dev. Cell* 49, 574-589.e5. 10.1016/j.devcel.2019.03.029 31006650 PMC6542281

[DMM052214C130] Zhao, Y. G., Codogno, P. and Zhang, H. (2021). Machinery, regulation and pathophysiological implications of autophagosome maturation. *Nat. Rev. Mol. Cell Biol.* 22, 733-750. 10.1038/s41580-021-00392-4 34302147 PMC8300085

[DMM052214C131] Zhou, F., Rasmussen, A., Lee, S. and Agaisse, H. (2013). The UPD3 cytokine couples environmental challenge and intestinal stem cell division through modulation of JAK/STAT signaling in the stem cell microenvironment. *Dev. Biol.* 373, 383-393. 10.1016/j.ydbio.2012.10.023 23110761 PMC3534909

[DMM052214C132] Zhou, J., Kryczek, I., Li, S., Li, X., Aguilar, A., Wei, S., Grove, S., Vatan, L., Yu, J., Yan, Y. et al. (2021). The ubiquitin ligase MDM2 sustains STAT5 stability to control T cell-mediated antitumor immunity. *Nat. Immunol.* 22, 460-470. 10.1038/s41590-021-00888-3 33767425 PMC8026726

