# Supplementary material for: Autophagy inhibition in intestinal stem cells favors enteroendocrine cell differentiation through Stat92E activity
Source: Dis Model Mech. 2025 Dec 29;18(12):dmm052214. doi: 10.1242/dmm.052214 (PMC12805648; doi:10.1242/dmm.052214)
Supplement: Supplementary information [file dmm-18-052214-s1.pdf]

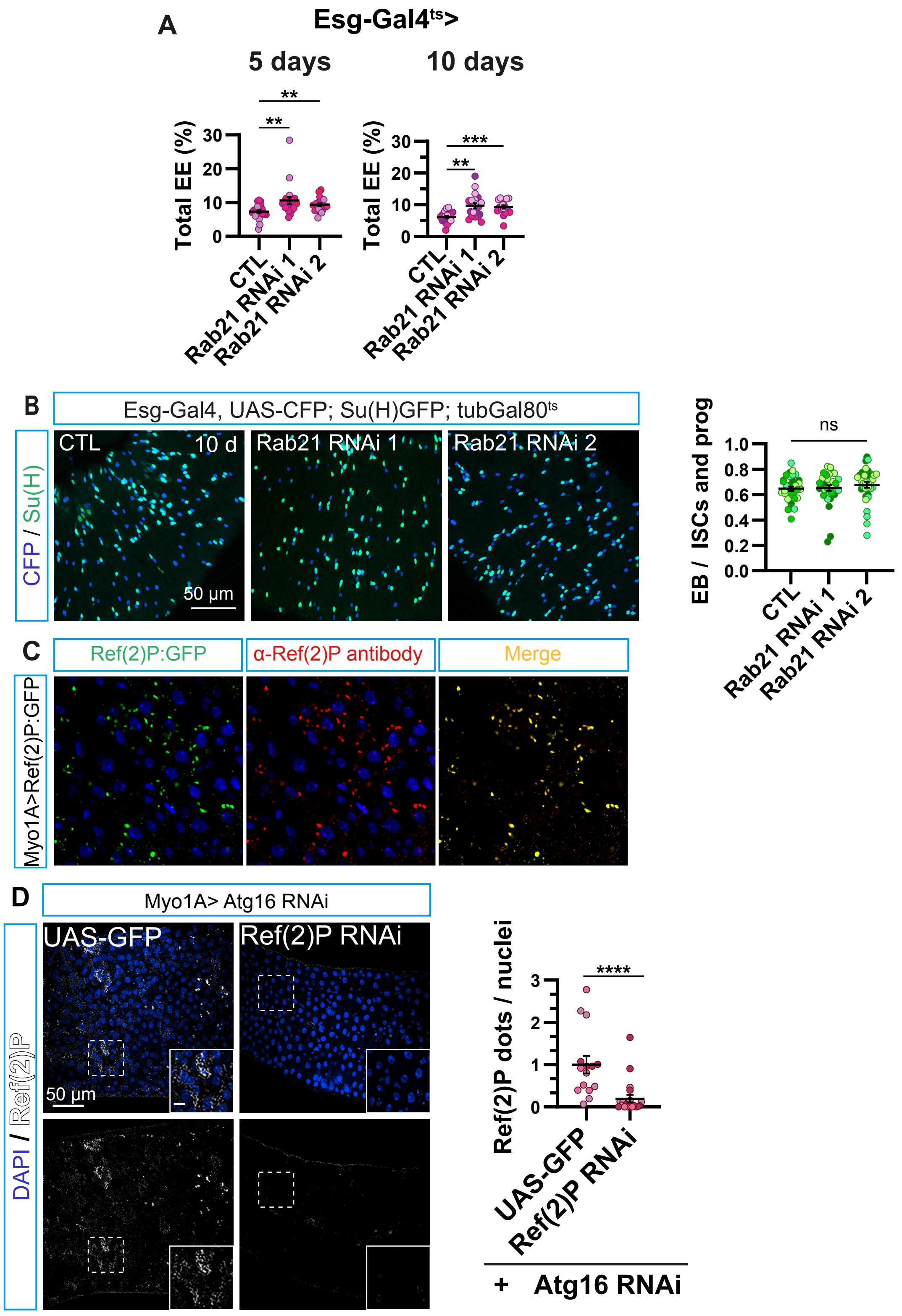

Fig. S1. Rab21 depletion in intestinal stem cells and progenitor cells affects the total number of enteroendocrine cells without affecting enteroblasts.

(A) Quantification of the percentage of total Pros$^+$ enteroendocrine cells over total cells (DAPI$^+$) related to Figure 1B, n ≥ 17 guts.

(B) Adult *Drosophila* posterior midgut from Esg-Gal4, UAS-CFP, Su(H)-GBE-GFP, tubGal80^{ts} driver expressing UAS-LacZ (CTL), RAB21 RNAi 1 or 2 for 10 days, in intestinal stem cells and progenitors. Representative maximal projections. CFP labels ISCs and progenitor cells (blue). GFP marks enteroblasts (EB). Scale bar 50 µm. The graph represents the quantification of the ratio of the number of GFP+ EB over the number of CFP$^+$ ISCs and progenitors, n ≥ 32 guts.

(C) Validation of Ref(2)P antibody by overexpressing UAS-Ref(2)P:GFP specifically in enterocytes of adult *Drosophila* posterior midgut with the Myo1A-Gal4, tubGal80^{ts} driver. Representative maximal projections. GFP marks overexpressed Ref(2)P proteins (green) which are labeled with the anti-Ref(2)P antibody (Red).

Disease Models & Mechanisms • Supplementary information

(D) Validation of Ref(2)P antibody by knocking-down Ref(2)P accumulation induced by Atg16 depletion in enterocytes of adult *Drosophila* posterior midgut. Myo1A-Gal4, tubGal80^{ts} driver was used to co-express Atg16 RNAi with UAS-GFP (CTL) or UAS-Ref(2)P RNAi. Representative maximal projections. Ref(2)P proteins are labeled with Ref(2)P antibody (white) and DAPI stains nuclei (blue). Quantifications of Ref(2)p dots per cells normalized to the CTL.

Data information: (A-B and D) N = three independent experiments from three independent crosses. (C) N = Two independent experiments from one independent cross. Quantifications represent the mean ± SEM. Each dot represents an intestine. The statistical tests used were: (A) unpaired t-tests. (B) Kruskal-Wallis test followed by Dunn's comparison test. * $p < 0.05$, *** $p < 0.001$, **** $p < 0.0001$, ns non-significant $p > 0.05$.

Disease Models & Mechanisms • Supplementary information

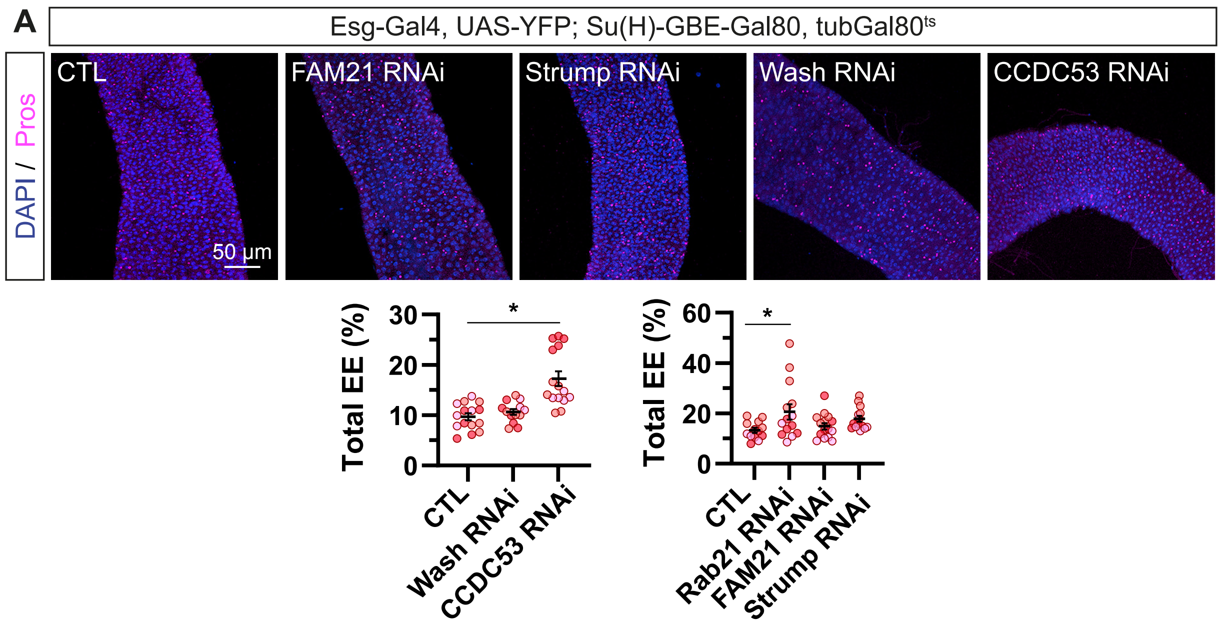

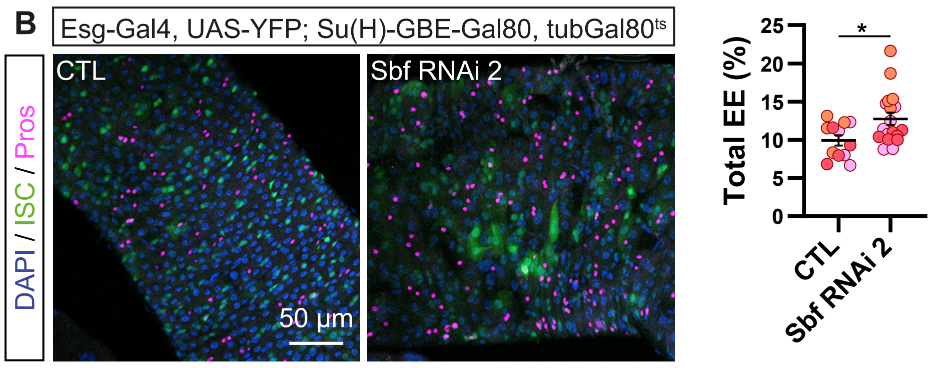

Fig. S2. Targeting Sbf with a different RNAi phenocopy the increase in enteroendocrine cells, while the knockdown of most of the Wash complex subunits does not.

(A) Adult *Drosophila* posterior midgut from Esg-Gal4, UAS-2xYFP; Su(H)-GBE-Gal80, tubGal80^{ts} driver expressing UAS-LacZ (CTL), Rab21 RNAi 2 or RNAis against subunits of the Wash complex, Wash, CCDC53, FAM21 or Strump for 10 days in intestinal stem cells. Prospero antibody marks enteroendocrine cells (magenta), and DAPI stains nuclei. Representative maximal projections. Scale bar 50 µm. Graphs represent the quantification of the percentage of Pros$^+$ mature enteroendocrine cells over total cells (DAPI$^+$), n ≥ 13 guts.

(B) Adult *Drosophila* posterior midgut from Esg-Gal4, UAS-2xYFP; Su(H)-GBE-Gal80, tubGal80^{ts} driver expressing UAS-LacZ (CTL), Sbf RNAi 2 for 10 days in intestinal stem cells. Prospero antibody marks enteroendocrine cells (magenta), and DAPI stains nuclei. Representative maximal projections. Scale bar 50 µm. The graph represents the quantification of the percentage of Pros$^+$ mature enteroendocrine cells over total cells (DAPI$^+$), n ≥ 12 guts.

Data information: N = three independent experiments from three independent crosses. Quantifications represent the mean ± SEM. Each dot represents an intestine. The Kruskal-Wallis test was used, followed by Dunn's comparison tests. * $p < 0.05$, *** $p < 0.001$, **** $p < 0.0001$, ns non-significant $p > 0.05$.

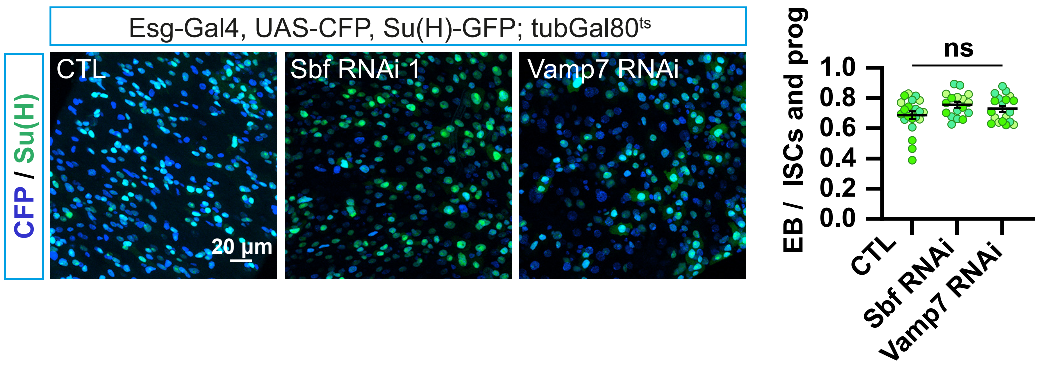

Fig. S3. Similarly to Rab21, Sbf or Vamp7 depletion in intestinal stem cells and progenitor cells does not affect the number of enteroblasts.

Adult *Drosophila* posterior midgut from Esg-Gal4, UAS-CFP, Su(H)-GBE-GFP, tubGal80^{ts} driver expressing UAS-LacZ (CTL), Sbf RNAi 1 or Vamp7 RNAi for 10 days, in intestinal stem cells and progenitors. Representative maximal projections. CFP labels ISCs and progenitor cells (blue). GFP marks enteroblasts (EB). Scale bar 20 µm. The graph represents the quantification of the ratio of the number of GFP+ EB over the number of CFP^{+} ISCs and progenitors, $n \geq 17$ guts.

Data information: N = three independent experiments from three independent crosses. Quantifications represent the mean ± SEM. Each dot represents an intestine. The Kruskal-Wallis test was used, followed by Dunn's comparison tests. * $p < 0.05$, *** $p < 0.001$, **** $p < 0.0001$, ns non-significant $p > 0.05$.

Disease Models & Mechanisms • Supplementary information

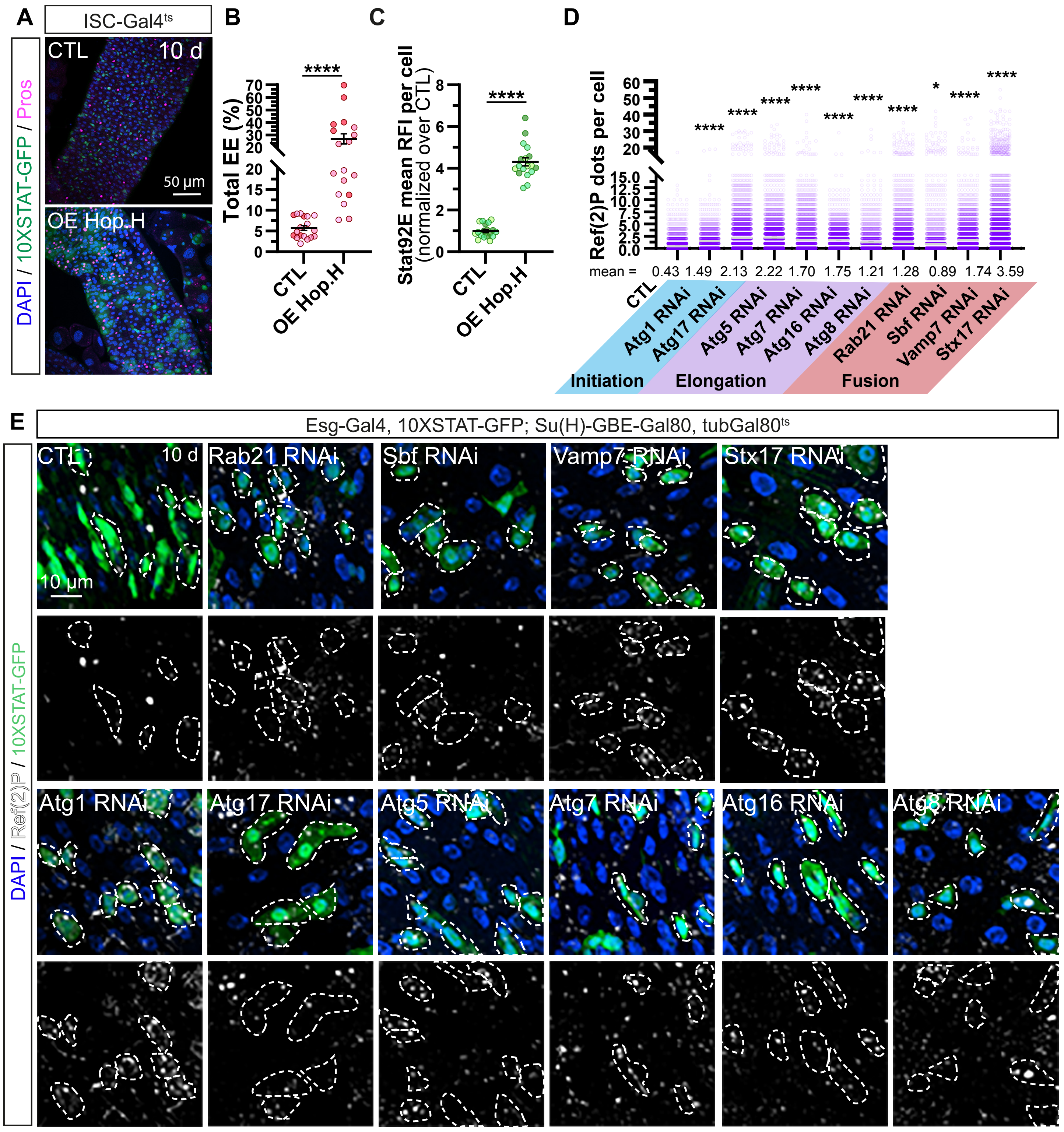

Fig. S4. Intrinsic overactivation of JAK-STAT by a constitutively active form of Hop strongly increases both enteroendocrine cells and Stat92E activity.

(A-E) Adult *Drosophila* posterior midgut from Esg-Gal4, 10XSTAT-GFP; Su(H)-GBE-Gal80, TubGal80^{ts} driver expressing UAS-mCD8:RFP (CTL) or RNAis against core Atg genes involved at different stages of autophagy or UAS-Hop.H for 10 days, in intestinal stem cells. (A) Representative maximal projections. 10XSTAT-GFP labels cells with active Stat92E (green). Prospero antibody marks enteroendocrine cells (magenta), and DAPI stains nuclei. Scale bar 50 µm. Quantification of (B) the percentage of total Pros$^+$ enteroendocrine cells over total cells (DAPI$^+$), n ≥ 18 guts, and (C) mean per intestine of 10XSTAT-GFP RFI per cell normalized to the control, RFI (relative fluorescence intensity), n ≥ 18 guts. (D–E) Related to Figure 6A. (D) Quantification of Re(2)P dots per 10XSTAT-GFP$^+$ ISC and progenitors, n ≥ 14 guts, n ≥ 1790 cells. (E) Representative maximal projections. 10XSTAT-GFP labels cells with active Stat92E (green). Ref(2)P antibody marks autophagosome cargos (white), and DAPI stains nuclei. Magnification of Ref(2)P dots in 10XSTAT-GFP$^+$ cells (dash lines). Scale bar 10 µm.

Data information: N = 4 independent experiments from four independent crosses. Quantifications represent the mean ± SEM. (A) Each dot represents Ref(2)P puncta per cell. (B) Each dot represents an intestine. The Kruskal-Wallis test was used, followed by Dunn's comparison tests. * $p<0.05$, *** $p < 0.001$, **** $p < 0.0001$, ns non-significant $p > 0.05$.

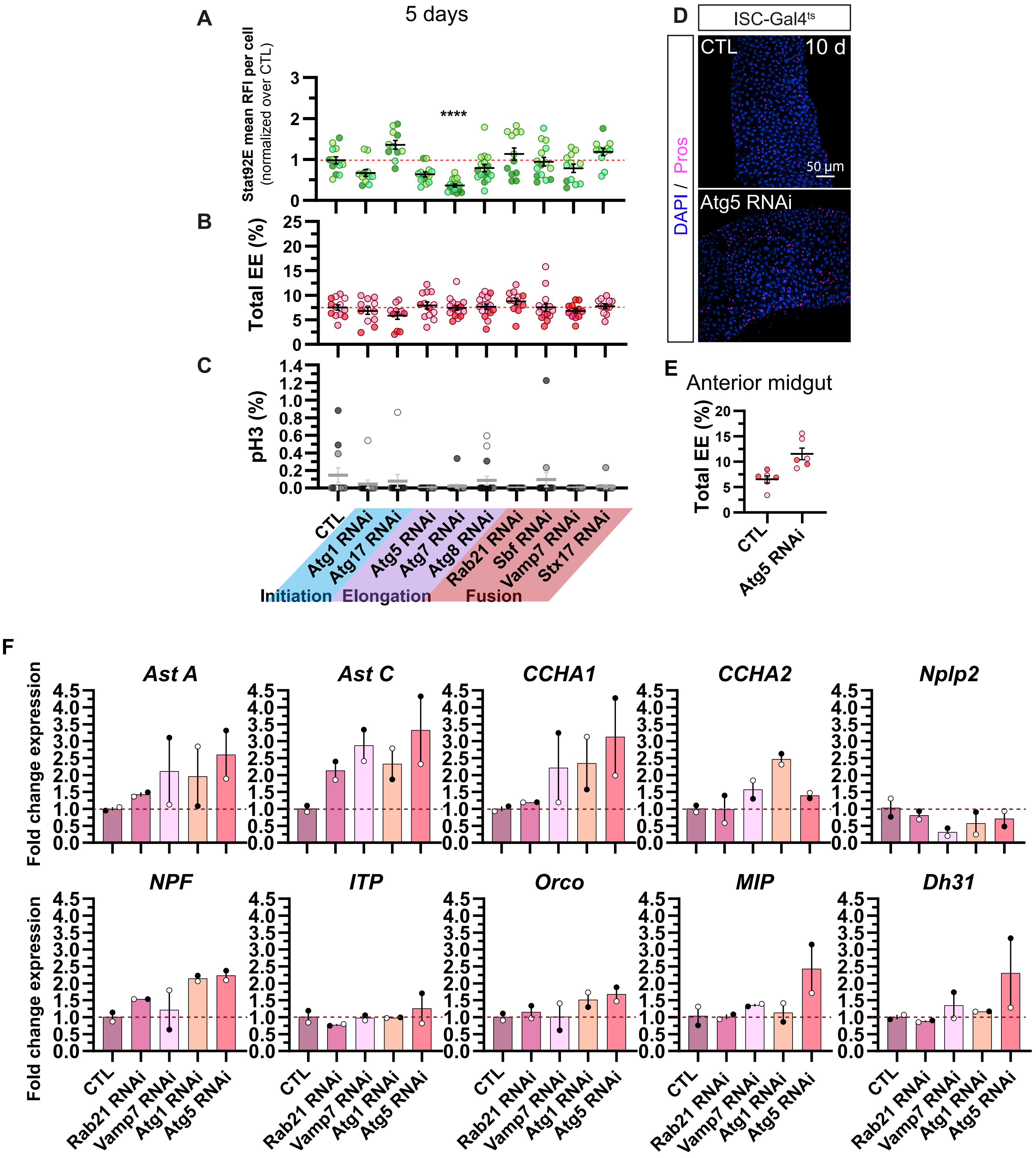

Fig. S5. The increase in enteroendocrine cell population associated with autophagy deficiency appears to extend beyond the posterior midgut and affects specific subtypes after 10 days of RNAi depletion.

(A-C) Adult *Drosophila* posterior midgut from Esg-Gal4, 10XSTAT-GFP; Su(H)-GBE-Gal80, TubGal80^{ts} driver expressing UAS-mCD8:RFP (CTL) or RNAis against core Atg genes involved at different stages of autophagy for 5 days, in intestinal stem cells. Quantification of (A) mean per intestine of 10XSTAT-GFP RFI

Disease Models & Mechanisms • Supplementary information

per cell normalized to the control, RFI (relative fluorescence intensity), (B) the percentage of total Pros$^+$ enteroendocrine cells over total cells (DAPI$^+$), n ≥ 12 guts, (C) the percentage of total pH3$^+$ mitotic cells over total cells (DAPI$^+$), n ≥ 12 guts (D-E) Adult *Drosophila* anterior midgut from Esg-Gal4, UAS-2XYFP; Su(H)-GBE-Gal80, TubGal80^{ts} driver expressing UAS-LacZ (CTL) or Atg5 RNAi for 10 days, in intestinal stem cells. (D) Representative maximal projections. Prospero antibody marks enteroendocrine cells (magenta), and DAPI stains nuclei. Scale bar 50 µm. (E) Quantification of the percentage of total Pros$^+$ enteroendocrine cells over total cells (DAPI$^+$), n = 6 guts. (F) Relative enteroendocrine subtype mRNA expression of adult *Drosophila* midgut from Esg-Gal4, UAS-2XYFP; Su(H)-GBE-Gal80, TubGal80^{ts} driver expressing UAS-LacZ (CTL) or Rab21, Vamp7, Atg1 or Atg5 RNAi for 10 days, in intestinal stem cells. Data information: (A-C) N = 3 independent experiments from 2 independent crosses, except for Rab21 RNAi. (D-F) N = 2 independent experiments from 2 independent crosses. Quantifications represent the mean ± SEM. Each dot represents an intestine. The Kruskal-Wallis test was used, followed by Dunn's comparison tests. * $p<0.05$, *** $p < 0.001$, **** $p < 0.0001$, ns non-significant $p > 0.05$.

Table S1. Reagents and resources.

Reagent or Resource	Source		Reference
***Drosophila* lines: Transgenes**			
UAS-Rab21 RNAi 1	VDRC	32941	Jean et al, 2015[37]
UAS-Rab21 RNAi 2	VDRC	109991	Jean et al, 2015[37]
UAS-Sbf RNAi 1	VDRC	22317	Jean et al, 2015[37]
UAS-Sbf RNAi 2	Bloomington	32419	Jean et al, 2015[37]
UAS-Stx17 RNAi	Bloomington	25896	Zang et al, 2019[20]
UAS-Vamp7 RNAi	NIG	1599R-1	Jean et al, 2015[37]
UAS-Atg1 RNAi	VDRC	16133	Zang et al, 2019[20]
UAS-Atg5 RNAi	VDRC	104461	Kiral et al, 2020[124]
UAS-Atg7 RNAi	VDRC	45558	Nagai et al, 2021[125]
UAS-Atg8 RNAi	Bloomington	34340	Zang et al, 2019[20]
UAS-Atg16 RNAi	Bloomington	34358	Zang et al, 2019[20]
UAS-Atg17 RNAi	Bloomington	36918	Wang et al, 2019[126]
UAS-Wash RNAi	VDRC	39769	Nassari et al, 2022[39]
UAS-CCDC53 RNAi	VDRC	28008	Nassari et al, 2022[39]
UAS-Strump RNAi	Bloomington	51906	Nassari et al, 2022[39]
UAS-FAM21 RNAi	Bloomington	50571	Nassari et al, 2022[39]
UAS-VPS26 RNAi	Bloomington	38937	Nassari et al, 2022[39]
UAS-Upd1 RNAi	Bloomington	28722	Rajan & Perrimon, 2012[83]
UAS-Upd2 RNAi	Bloomington	33988	Rajan & Perrimon, 2012[83]
UAS-Dome RNAi	Bloomington	32860	Rajan & Perrimon, 2012[83]
UAS-Hop RNAi	Bloomington	31319	Sanchez et al, 2019[84]
UAS-Stat92E RNAi	Bloomington	33637	Rajan & Perrimon, 2012[83]
UAS-Stg RNAi	Bloomington	29556	Weber & Mlodzik, 2017[127]
UAS-EGFR RNAi	Bloomington	25781	Zang et al, 2019[20]
UAS-Scute RNAi	Bloomington	26206	Chen et al, 2018[47]

Disease Models & Mechanisms • Supplementary information

UAS-Atg1	Bloomington	51655	Ulgerhait et al, 2014[128]
UAS-Delta-GFP	Bloomington	8611	Beebe et al, 2009[80]
UAS-Hop.H	Bloomington	79033	Harrison et al, 1995[129]
UAS-Stat92E	Erika Bach		Ekas et al, 2010[88]
UAS-Stat92E; UAS-Stat92E$^{\Delta N\Delta C}$, refer as UAS-Stat92E.CA	Erika Bach		Ekas et al, 2010[88]
UAS-Sox21a	Bloomington	68156	Chen et al, 2016[97]
UAS-Ref(2)P RNAi	Bloomington	36111	Gumeni et al., 2021[130]
UAS-Ref(2)P::GFP	Bloomington	605356	
UAS-LacZ	Bloomington	1776	
UAS-GFP	Bloomington	35786	
UAS-mCD8:RFP	Bloomington	27391	
10XSTAT-GFP	Bloomington	26197	
UAS-Rab21 RNAi 1, 10XSTAT-GFP	Nassari et al, 2022		
UAS-LacZ, 10XSTAT-GFP	Nassari et al, 2022		
UAS-Rab21 degenerate	Nassari et al, 2022		
***Drosophila* lines: Drivers**			
Esg-Gal4, UAS-GFP, TubGal80^{ts}/ CyO	Edgar B.		
Esg-Gal4, UAS-mCD8:GFP/ CyO; Gal80^{ts}	Biteau B.		
Esg-Gal4, UAS-YFP/ CyO; Su(H)-GBE-Gal80, tubGal80^{ts}/ TM3, Sb – ISC-Gal4^{TS}	Bardin A.		
Esg-Gal4, UAS-mCD8::GFP/ CyO; UAS-H2B::RFP, tubGal80GAL80^{TS}/ TM3,Ser – ReDDM	Dominguez M.		
Esg-Gal4, UAS-CFP, Su(H)-GFP/ CyO, tubGal80^{ts} / TM3, Sb	O'Brien L.		
{gbe-GAL80}ZH-2A; {mira -KDRT>-	Sokol N.		Buddika K. et al.,

Disease Models & Mechanisms • Supplementary information

dSTOP-KDRT>-GAL4}attP40, P{tubP-GAL80[ts]}20; {CG10116-KD.PEST}attP2) – ISC-KCKT^{ts} (ISC-intestinal-kickout-GAL4)		2021[131]
Myo1A-Gal4, tubgal80^{TS}	Edgar B	
Esg-Gal4, 10XSTAT-GFP/ CyO; GBE-Su(H)Gal80, tubGal80^{ts}/ TM3,Ser	This Paper	
Reagent or Resource	Source	Reference
**Antibodies**		
Anti-Prospero	DSHB M1RA	
Anti-phospho-histone H3 (Ser10)	Millipore 06-570	
Anti-Ref(2)P	Abcam ab178440	Wang et al, 2023[62]
Anti-discs large	DSHB 4F3	
Goat anti-Mouse IgG (H+L) Secondary Antibody, Alexa Fluor® 647 conjugate	Thermo Fisher Scientific	A21236
Goat anti-Rabbit IgG (H+L) Secondary Antibody, Alexa Fluor® 546 conjugate	Thermo Fisher Scientific	A11035
**Chemical reagents**		
Glycerol	Fisher Scientific	AAJ61059K2
Trizol	Invitrogen	15596026
Maxima First Strand cDNA Synthesis kit	Thermo Fisher Scientific	K1671
Luna universal qPCR mastermix	NEB	M3003
20 % Formaldehyde Aqueous Solution (Paraformaldehyde Aqueous Solution) EM Grade	Electron microscopy science	15713
DAPI	New England Biolabs	4083S
SlowFade™ Gold Antifade Mountant	Thermo Fisher Scientific	s36940
SlowFade™ Gold Antifade Mountant with DAPI	Thermo Fisher Scientific	S36938
Software		

Disease Models & Mechanisms • Supplementary information

Image J	https://fiji.sc/
CellProfiler	https://cellprofiler.org/releases
IMARIS	Oxford Instruments
Prism	GraphPad
Photoshop	Adobe
Illustrator	Adobe

Table S2. Primers used.

**Primers**	**Sequence**	**Primers**	**Sequence**
dAstA_F	GAGGTCTCGTCCCTACTCCTTC	*dAstA_R*	GATCTCGTTGTCCTGGTCGT
dDH31-F	GCCAATCCAATGGAGGATAC	*dDH31-R*	GTATGATGGTGCGTCCAAAG
dMIP-F	GTAAGCGCGAACCCACATG	*dMIP-R*	CCTGTGCTACGGCGATTCTC
dNPF-F	GTGACACCGTTGCGCTTTCC	*dNPF-R*	GCCACACAGGCAACCAGGAT
dOrco-F	GTGCTCCTGGCGGTGGTATC	*dOrco-R*	CGGCGATGTGGAAAGGTCGT
dCCHa1-F	ACTGACGTCGGACAATTTGC	*dCCHa1-R*	ACACGAATGTCCGTATTCCA
dCCHa2-F	AAACAGCAACAGCAGCAAAC	*dCCHa2-R*	AGGACCACGGTGCAGATAAC
dASTC-F	ATATGCCAGCCCAGGCAATC	*dASTC-R*	CGGGCAGCCGATAAAGTTCA
dNplp2-F	CTTACCAAGGCCCAGGGTGA	*dNplp2-R*	TGCGGCTGGTGCCTGAAGAA
dITP-F	GCAAGGGCATCTTCAACAAG	*dITP-R*	GTTGGATATTTCCTCTTCGGG

Disease Models & Mechanisms • Supplementary information
